# Discovering probiotic microorganisms: *in vitro*, *in vivo*, genetic and omics approaches

**DOI:** 10.3389/fmicb.2015.00058

**Published:** 2015-02-17

**Authors:** Konstantinos Papadimitriou, Georgia Zoumpopoulou, Benoit Foligné, Voula Alexandraki, Maria Kazou, Bruno Pot, Effie Tsakalidou

**Affiliations:** ^1^Laboratory of Dairy Research, Department of Food Science and Human Nutrition, Agricultural University of Athens, AthensGreece; ^2^Bactéries Lactiques et Immunité des Muqueuses, Institut Pasteur de Lille, Centre d‘Infection et d’Immunité de Lille, Université Lille Nord de France, CNRS UMR8204, LilleFrance

**Keywords:** probiotics, screening, mechanism, *in vitro* model, *in vivo* model, omics, molecular marker, health claim

## Abstract

Over the past decades the food industry has been revolutionized toward the production of functional foods due to an increasing awareness of the consumers on the positive role of food in wellbeing and health. By definition probiotic foods must contain live microorganisms in adequate amounts so as to be beneficial for the consumer’s health. There are numerous probiotic foods marketed today and many probiotic strains are commercially available. However, the question that arises is how to determine the real probiotic potential of microorganisms. This is becoming increasingly important, as even a superficial search of the relevant literature reveals that the number of proclaimed probiotics is growing fast. While the vast majority of probiotic microorganisms are food-related or commensal bacteria that are often regarded as safe, probiotics from other sources are increasingly being reported raising possible regulatory and safety issues. Potential probiotics are selected after *in vitro* or *in vivo* assays by evaluating simple traits such as resistance to the acidic conditions of the stomach or bile resistance, or by assessing their impact on complicated host functions such as immune development, metabolic function or gut–brain interaction. While final human clinical trials are considered mandatory for communicating health benefits, rather few strains with positive studies have been able to convince legal authorities with these health claims. Consequently, concern has been raised about the validity of the workflows currently used to characterize probiotics. In this review we will present an overview of the most common assays employed in screening for probiotics, highlighting the potential strengths and limitations of these approaches. Furthermore, we will focus on how the advent of omics technologies has reshaped our understanding of the biology of probiotics, allowing the exploration of novel routes for screening and studying such microorganisms.

## INTRODUCTION

Probiotic research faces new challenges. Currently, there is an increased legislative pressure, in both the EU and the USA, to strictly limit the health communications of probiotics. While a more strict regulation is of course not a major problem as such, it may considerably hamper the release of new probiotic strains and applications. This is especially worrisome, as the boost of the metagenomics research efforts starts to pay off by creating numerous new and interesting working hypothesis for microbiota manipulation in maintaining and restoring health. Results of this research, for the first time, allow to avoid the tedious screening of large numbers of strains and to identify potential new health promoting bacteria from the comparison of population with different health status (lean versus obese, allergic versus non-allergic, etc.). Several new applications have been suggested already either via a supplemented diet or via a pharmaceutical approach. Improving *Faecalibacterium prausnitzii* levels has been suggested as beneficial for inflammatory bowel disease (IBD) patients (Miquel et al., 2013), the use of *Akkermansia muciniphila* has recently been patented for treating metabolic disorders (Cani et al., 2014) and the role of dietary bioactive proteins and peptides in autism spectrum disorders may also result in new probiotic strains in the market (Siniscalco and Antonucci, 2013). The legislative framework today is not ready to cope with these new applications. Approval will require a full analysis of the mechanism of action. A full inventory of the risks will have to be determined in different populations, at different doses and using different delivery modes and matrices. The research approaches presented in this review aim to assist in this process. While many of the *in vitro* models described here may seem outdated, they are still used for cost and ethical reasons. The use of new molecular omics-based technologies is increasing fast and it will most probably replace traditional screening methods. Omics technologies may also turn out to be very effective in the follow-up analysis of probiotic candidate strains resulting from *in vitro* and/or *in vivo* screening with current methodologies. Genome sequencing, as an example, will allow to quickly detect and eliminate strains that pose a potential risk, through the presence of antibiotic resistance or virulence genes. The new research approaches will also facilitate the analysis and description of functional mechanisms, facilitating the construction of health claim or pharmaceutical dossiers. Another consequence of this focus on mechanisms might be that live microorganisms will no longer be necessary, but they will be replaced by the active ingredients or metabolites identified as the active compound. This might cause a shift for certain applications from the food to the pharma area. However, it remains to be shown if this shift will also result in active ingredients that have no side effects, as it is currently expected from probiotics. The use of the models and research strategies described in this review, alone or in combination, may help to answer that type of questions.

## DOCUMENTING PROBIOTICS WITH *IN VITRO* ASSAYS

### SURVIVING STRESS WITHIN THE HOST

Since the early days of probiotics research, *in vitro* screening of probiotics was a preferable choice due to the simplicity and the low cost of such approaches (**Table 1**). Even though some of these tests may seem outdated they are still in use and they can be found in recent reports. Perhaps the most important advantage of *in vitro* assays is their ability to screen multiple strains simultaneously.

**Table 1:** *In vitro* assays employed during screening for novel probiotic strains.

Probiotic property	Assays	Representative references
Surviving stress within the host	Low pH and bile (e.g., artificial gastric and pancreatic juices and GIT simulators)	Conway et al. (1987), Molly et al. (1994), Marteau et al. (1997), Charteris et al. (1998), Minekus et al. (1999), Mainville et al. (2005), Lavermicocca et al. (2008), Bove et al. (2012), Cook et al. (2012), Van den Abbeele et al. (2012)
Safety assays	Antibiotic resistance	Delgado et al. (2007), Argyri et al. (2013), Pisano et al. (2014)
	Hemolytic activity	Pisano et al. (2014)
	Adhesion to mammalian cells	Harty et al. (1994)
	Production of enzymes (e.g., glycosidases)	Oakey et al. (1995), Bernardeau et al. (2006)
	Production of toxins (e.g., cytolysins)	Tan et al. (2013)
	Production of biogenic amines	Halász et al. (1994), Bover-Cid and Holzapfel (1999)
Colonization of the host	Cell surface hydrophobicity	Ouwehand et al. (1999), Vinderola et al. (2004), Kaushik et al. (2009), Jena et al. (2013), García-Cayuela et al. (2014)
	Adhesion to mucus (e.g., adhesion to mucin, enzymatic activity of GAPDH)	Kirjavainen et al. (1998), Laparra and Sanz (2009), Ferreira et al. (2011), Kinoshita et al. (2013)
	Auto-aggregation screening	Collado et al. (2008), Bao et al. (2010), Malik et al. (2013), Botta et al. (2014)
	Adhesion to intestinal epithelium (e.g., cell-lines, tissue fragments and whole tissue models)	Vesterlund et al. (2005), Muller et al. (2009), Tassell and Miller (2011)
Antimicrobial assays	Production of antimicrobial metabolites such as organic acids and bacteriocins (e.g., simple inhibition tests, turbidometric assays, bioluminescence assay, streak methods)	Tagg and McGiven (1971), Jacobsen et al. (1999), Strus et al. (2005), Lahtinen et al. (2007), Parkes et al. (2009), Guo et al. (2010), Haukioja (2010), Lahteinen et al. (2010), Chenoll et al. (2011), Cresci et al. (2013), Kechaou et al. (2013), Al Kassaa et al. (2014), Coman et al. (2014)
	Co-aggregation with pathogens	Collado et al. (2007), Bao et al. (2010)
	Enhancement of intestinal barrier function (e.g., TER measurement, immunofluorescence of tight junction protein antibodies, tight junctional protein phosphorylation)	Resta-Lenert and Barrett (2003), Zyrek et al. (2007), Ewaschuk et al. (2008)
Immunomodulation	Bacterial translocation in the GIT	Corthesy et al. (2007)
	Co-culture models mimicking *in vivo* situation (e.g., co-culture models or three component models with epithelium cells, immune cells and bacteria)	Borchers et al. (2009), Cencic and Langerholc (2010)
	Interaction of host immune system with bacterial compounds (e.g., lipoteichoic acids and peptidoglycan)	Miettinen et al. (1998), Corthesy et al. (2007), Steinberg et al. (2014)
	Regulation of epithelial tight junctions	McKay et al. (1997)
	Anti-inflammatory immune-stimulating properties (e.g., alleviation of IBD and allergic symptoms)	Fujiwara et al. (2004), Smits et al. (2005), Foligne et al. (2007)
	β-Hexosaminidase release assay (alleviation of allergic reactions)	Kim et al. (2013)
Cardiovasular diseases	Deconjugation of bile salts (e.g., BSH activity)	Dashkevicz and Feighner (1989), Smet et al. (1994), Zheng et al. (2013)
	Conversion of cholesterol to coprostanol	Lye et al. (2010)
	Peptides from bacterial metabolism with ACE inhibitory activity	Papadimitriou et al. (2007)
Anticancer	Ames test	Burns and Rowland (2004)
	Comet assay	Pool-Zobel et al. (1996)
	Nitrosamine degrading assay	Duangjitcharoen et al. (2014)
	Preventing colon cancer cell invasion	Choi et al. (2006)
	Induction of apoptosis of cancer cells	Castro et al. (2010)
	Binding to mutagenic compounds (HCAs)	Faridnia et al. (2010)
	Removal of toxins and toxic metals	Halttunen et al. (2007); Nybom et al. (2008)
	Bacterial fermentation and production of SCFAs	Boffa et al. (1992), Mariadason et al. (1999), Sakata et al. (2003), Cousin et al. (2012)
Additional health benefits	β-Galactosidase activity	Hughes and Hoover (1995)
	Production of vitamins	Pompei et al. (2007)
	Linolenic acid test	Kullisaar et al. (2002)
	Oxalate-degradation	Campieri et al. (2001)

According to current definitions, probiotics should be viable, even though sometimes dead microbial cells can also exert health benefits (Salminen et al., 1999). It is also recommended that probiotics must be able to reach the desired body niches alive. An initial screening of strains based on various stress tolerance assays is of utmost importance (Upadrasta et al., 2011), especially for non-encapsulated strains directly used in food. Hence, appropriate *in vitro* tests have been adopted to select strains based on their ability to survive transit through the different compartments of the gastrointestinal tract (GIT; Joint FAO/WHO Working Group, 2002).

Survival of potential probiotic bacteria under simulated GIT conditions has been extensively studied over the last decades and strain-specific differences are marked throughout the literature. Following ingestion, probiotics first encounter the harsh conditions of the stomach and they must be able to survive under the extreme acidic conditions and the activities of the digestive enzymes. The pH of the stomach is known to fluctuate from 1–2 up to 4–5 after food consumption but most *in vitro* assays have been developed to select strains that withstand extreme low pH values. Most conventional methodologies include experiments studying the survival of strains in buffers with no nutrients like PBS or modified growth media, all adjusted to low pH. Similar experiments have not been reported for high values of pH, mimicking the slightly alkaline conditions of the small intestine, perhaps reflecting the notion that most probiotic strains are resistant to alkaline conditions. Acid tolerance tests are among the simplest tests that can be performed, allowing the routine screening of large numbers of strains. However, given the unrealistic harsh pH conditions employed during these tests, they may result in the loss of relatively acid sensitive probiotic candidates. For example, acid sensitive strains could be protected from the acid challenge of the stomach due to the buffering properties of food vehicles or specific food ingredients. Furthermore, the strains are most often challenged as pure cultures in either log or stationary phase, while in reality, probiotics are delivered to the host already stressed due to extended fermentation periods, food processing conditions, and storage. This pre-stressed state of probiotics may lead either to enhanced or diminished stress resistance during passage through the host, a property that may be species or even strain dependent.

Except for these simplified survival tests, artificial gastric as well as pancreatic juices have been developed to better represent the *in vivo* conditions (Charteris et al., 1998). The survival in true gastric juice obtained from human individuals has been reported (Conway et al., 1987). Generally, synthetic gastric and pancreatic juices include the enzymes pepsin and pancreatin, respectively, and controlled incubation of strains in these juices have been investigated to mimic the time spent by probiotics in the upper and the lower GIT (Lavermicocca et al., 2008). Bile secreted in the small intestine reduces the survival of bacteria by disrupting the cell membrane, by inducing protein misfolding and denaturation and by damaging DNA. Bile salt hydrolase (BSH) is an enzyme that hydrolyses the amino acids of conjugated bile salts (glycine or taurine), reducing their toxicity. Tolerance to bile salt concentrations between 0.15 and 0.5% has been recommended for probiotics, which is in the range of the physiological concentrations met in the GIT (Gorbach and Goldin, 1992). Again, bile tolerance assays may be easy to perform, but they may not particularly facilitate the reliable selection of probiotics, for several reasons. For example, in most cases strains are separately studied for acid or bile tolerance, despite the fact that these two stresses are actually sequential during passage through the GIT, increasing the stress pressure. The use of non-human bile may also raise some questions, as bovine or porcine bile do not have the same impact on microorganisms as human bile (Begley et al., 2005).

The need for more elaborate *in vitro* assays for testing the fate of probiotic strains in the GIT led to the development of several GIT simulators. More precisely, a multi-compartmental dynamic computer-controlled model simulating the stomach and the small intestine (Minekus et al., 1999) has been used to quantify the survival of lactic acid bacteria (LAB) and the data obtained correlated well with those obtained from human subjects (Marteau et al., 1997). In other cases, *in vitro* systems reproduce not only the conditions of the stomach and the small intestine but also those occurring in the oral cavity using an oro-gastric-intestinal (OGI) system (Bove et al., 2012). The simulator of the human intestinal microbial ecosystem (SHIME) was developed by inoculating human fecal material in a fermenter-based simulator to establish a GIT-like microbial population (Molly et al., 1994). Experiments with SHIME revealed similar survival rates of microorganisms to those obtained with *in vivo* tests (Cook et al., 2012). A modification of the SHIME system involved the incorporation of a mucosal environment in the SHIME model, resulting in a more representative colonization ability for the test strains (Van den Abbeele et al., 2012). Another system that relied on two separate fermenters was designed to better simulate the physiological events of ingestion and digestion in the upper GIT. Using this system, it was possible to investigate the survival of probiotics through more realistic pH values, i.e., those that prevail prior, during and after a meal (Mainville et al., 2005). Obviously, GIT simulators offer many advantages over independent *in vitro* tests and the selection of probiotic strains using these systems may be more reliable. However, such simulators do not allow rapid screening of multiple strains and they may be relatively expensive to maintain and operate. Today advancements in encapsulation technology allow the targeted delivery of probiotic strains to different compartments of the GIT in an active state irrespectively of their stress robustness.

### SAFETY ASSAYS

Another important aspect in selecting probiotic strains is their safety status. While in Europe QPS regulation has identified the microorganisms that can be safely used in foods, there might be some safety aspects that may need to be evaluated before commercial probiotic cultures are put on the market (Joint FAO/WHO Working Group, 2002). Laboratory tests applied for the safety evaluation of probiotic cultures include *in vitro* assays examining different intrinsic properties of the strains. Initially, the minimum inhibitory concentrations (MICs) for the most relevant antibiotics is usually determined and evaluated using protocols given by EFSA (2008). The microdilution-broth test performed on 96-well microplates (Argyri et al., 2013), the disk-diffusing method (Pisano et al., 2014) and ready-to-use commercial kits (Delgado et al., 2007) have been applied to specify MIC values of known antibiotics for potential probiotic strains in many cases. Hemolytic activity is also examined (Joint FAO/WHO Working Group, 2002). Clear zones of hydrolysis, partial hydrolysis or no reaction around the streaking of strains on blood agar plates indicate the hemolytic ability of probiotics (Pisano et al., 2014). *In vitro* tests of pathogenic traits concern the ability of bacteria to bind to mammalian cells such as platelets, which is coupled with their binding to fibronectin, fibrinogen and collagen (Harty et al., 1994). The production of certain enzymes (e.g., glycosidases, proteases and gelatinases) is also perceived as a potential pathogenicity trait (Oakey et al., 1995; Bernardeau et al., 2006). Strains should be tested with appropriate *in vitro* assays for the production of known human toxins (e.g., cytolysins; Tan et al., 2013). Biogenic amines are usually generated by decarboxylation of the corresponding amino acids through substrate-specific decarboxylases of bacteria. Assays performed on solid media are based on the pH change of the medium after bacterial growth, corresponding to positive decarboxylase activity (Bover-Cid and Holzapfel, 1999). Quantitative analysis of biogenic amines is generally accomplished by chromatography using amino acid analyzers (Halász et al., 1994). *In vitro* safety tests are generally very useful to identify and exclude clean-cut cases of pathogenic strains from being used as probiotics. For example, a hemolytic or a toxin producing strain can be easily identified and excluded from further analysis. The problem with the *in vitro* safety assays concerns the identification of false negative strains. A virulence trait may be simply non-active under the specific conditions of the assay and thus remain undetectable (e.g., a toxin that may be down-regulated *in vitro*). Virulence is a complex phenomenon that sometimes needs an active interaction with the host to be triggered and for this reason *in vivo* models may be more appropriate. The screening of the bacterial genome for the presence of virulence and resistance genes (see below) is also a way to predict the possibility of non-expressed safety risk factors.

### COLONIZATION OF THE HOST AS A PREREQUISITE TO EXERT CERTAIN HEALTH BENEFITS

Although research in the probiotic area has considerably progressed the last decades, the correlation of specific cultures with specific health claims is still ambiguous. In a relatively limited number of cases specific *in vitro* assays have been devised to investigate the protective or therapeutic role of probiotic candidates against certain diseases. The simplest application is the competition of the probiotics with potential pathogens for resources and space in the GIT. Adherence to mucus and epithelial cells is still considered a controversial topic in probiotics research. On the one hand it is a desirable probiotic trait, as it facilitates colonization of the host and antagonism against pathogens, but on the other hand it is considered as a risk for translocation. The latter might be especially important in highly sensitive populations of immune depressed patients where probiotic applications are often considered (Sanders et al., 2010).

The hydrophobicity phenotype of bacterial cell surface is related to their adhesive capacity and colonization of the gut (Ouwehand et al., 1999). Generally, cell surface hydrophobicity is determined according to the capacity of the bacteria to partition into hydrocarbons (i.e., hexadecane, xylene, toluene; Kaushik et al., 2009; Jena et al., 2013), thus, reflecting the non-specific adhesion capability related to cell surface characteristics (García-Cayuela et al., 2014). Controversial results of hydrophobicity studies show that this feature might be questionable (Vinderola et al., 2004). In general, assessing the adhesive capacity of probiotic strains based on surface hydrophobicity is rather outdated.

Adhesion tests of probiotics to human intestinal mucus obtained from infants or healthy human feces have been performed (Kirjavainen et al., 1998; Ferreira et al., 2011). Moreover, high-throughput screening methods based on immobilized commercially available mucin have also been reported (Laparra and Sanz, 2009). Mucins are large glycoproteins that fortify intestinal mucosal surfaces forming a protective shield for the epithelial cells against harmful environmental conditions. Glyceraldehyde-3-phosphate dehydrogenase (GAPDH) expressed on the bacterial cell surface aid binding to human colonic mucin and the evaluation of this enzymatic activity has been reported as a simple screening method (Kinoshita et al., 2013). Alternatively, other studies have focused on the ability of probiotic bacteria to form cellular aggregates via self-aggregation (auto-aggregation; Del Re et al., 2000) by measuring absorbance of bacterial suspensions that are left standing for certain time intervals (Collado et al., 2008). Auto-aggregation capacity of LAB is correlated to their adhesion to different kind of host cells (Malik et al., 2013), and it is considered as a desirable characteristic for preliminary probiotic screening (Bao et al., 2010; Botta et al., 2014). Intestinal epithelial cell (IEC) lines are often presumed to better represent conditions in the tissues of the GIT (i.e., adhesion ability and colonization of probiotic strains). Several studies have been conducted using human epithelial cell lines (like HT-29, HT-29MTX, and Caco-2) to screen the adhesion of probiotic strains (Muller et al., 2009). In general, the *in vitro* testing of the adhesion potential is considered experimentally difficult. Reproducibility issues have been observed among laboratories due to the use of different variants of a given cell line and of high background levels (Kirjavainen et al., 1998). These tests can only yield rough indications of a strain’s potential to adhere *in vivo*. Additionally, resected intestinal tissue fragments have been used unprocessed or immobilized on microtitre plates for adhesion assays (Vesterlund et al., 2005). Finally, the whole tissue models consisting of the epithelial tissue with the mucus layer in the presence of commensal microbiota may allow the assessment of more complex adhesive interactions between probiotics and the host (Tassell and Miller, 2011).

Several molecules that are actively aiding the binding to host’s cells have been identified. The problem with the *in vitro* assays is their inability to recapture the actual conditions prevailing in the GIT. In most cases adhesion is studied for single strains thus in the absence of any additional microbiota that would mimic the gut microbiome. This is a very important drawback for most assays, since there is fierce competition for adhesion sites among the different microbes *in vivo*. The use of cancer cells is also a bit controversial since their extracellular matrix and surface properties may differ significantly from that of healthy IECs. Nevertheless, strains shown to adhere with high efficiency to human cells *in vitro* usually behaves in a similar manner *in vivo*.

### ANTIMICROBIAL ASSAYS

Another desired attribute is the production of antimicrobial compounds by probiotics. Perturbation of the GIT microbiome plays an important role in the pathophysiology of common gastrointestinal infectious diseases. Researchers have proposed that probiotics may prevent gastrointestinal disorders by maintaining homeostasis of the gut microbiome or by competitively inhibiting the growth of pathogens (Hickson, 2011). Selection of probiotic bacteria active against infectious diarrhea attributed to viruses (e.g., rotavirus and norovirus; Al Kassaa et al., 2014) or bacteria (e.g., *Escherichia coli, Salmonella* and *Campylobacter* sp.) or to *Clostridium difficile* infection (Parkes et al., 2009), is usually based on antimicrobial properties of probiotic strains. This is also the case for antibiotic-associated diarrhea (Cresci et al., 2013) and *Helicobacter pylori* infection (Chenoll et al., 2011), as well as for infections related to sites of the human body other than the GIT, such as the oral cavity (Haukioja, 2010), the upper respiratory tract (Kechaou et al., 2013), and the urogenital system (Strus et al., 2005). In addition to the production of known antimicrobial metabolites such as organic acids, probiotic bacteria may also produce specialized inhibitory agents, like bacteriocins. Target strains commonly include both Gram positive and Gram negative bacteria, as well as fungal strains, comprising of not only pathogenic bacteria but also strains representative of the predominant human GIT microbiota (Gagnon et al., 2011). In general, antagonistic activity is evaluated *in vitro* using simple inhibition tests performed on solid media. More precisely, the agar spot test (Jacobsen et al., 1999), the paper-disk diffusion assay (Guo et al., 2010), and the well diffusion assay (Tagg and McGiven, 1971) have been extensively used as methods for evaluating antimicrobial activity. Moreover, inhibitory effects of probiotic culture filtrates assessed by an automated turbidometric assay that monitors the ability of the indicator bacteria to grow has also been reported (Lahteinen et al., 2010). In some cases, bioluminescent indicator strains were also used to investigate the possible production of antimicrobial compounds from probiotic bacteria (Lahtinen et al., 2007). Assays as the cross-streak and the radial streak methods are comparatively more efficient in terms of examining the inhibitory properties of intact probiotic cells and not only properties attributed to their producing metabolites (Coman et al., 2014).

Antimicrobial ability of probiotics refers not only to the production of antimicrobial compounds or acids that affect luminal pH (Suardi et al., 2013), but also the competitive exclusion of pathogens. Probiotics may compete with pathogens for binding sites on the surface of the GIT. The *in vitro* adhesion assays mentioned earlier can be used to assess the binding competition between probiotic and pathogenic strains. In this context, bacterial aggregation between genetically distinct cells (co-aggregation) is of considerable importance. Thus, protective properties of probiotics against pathogen infections can also be evaluated through co-aggregation assays based not only on simple absorbance measurements but also on radiolabelling and fluorescence detection (Collado et al., 2007).

Antimicrobial properties of probiotics are also correlated with the enhancement of the intestinal barrier function (Mennigen and Bruewer, 2009). Barrier properties can be investigated by measuring trans-epithelial electrical resistance (TEER) before and after apical exposure of IEC lines to bacteria (Zyrek et al., 2007). Promotion of tight junction integrity is known to block paracellular transport of pathogenic bacteria. Alterations of tight junction proteins are examined *in vitro* either by immunofluorescence using specific antibodies (Ewaschuk et al., 2008), or by evaluating the capacity of probiotics to alter tight junctional protein phosphorylation (Resta-Lenert and Barrett, 2003).

Once more, the *in vitro* production of antimicrobial substances alone cannot provide us with important information about the probiotics application *in vivo*. We cannot know if the selected strain will be able to be incorporated in the microbiome and whether the conditions prevailing in the GIT will allow it to produce its antimicrobial(s) compounds at sufficient amounts to have an effect. Usually, probiotic strains produce more than one antimicrobial substance that may act synergistically, increasing the spectrum of targeted microorganisms. This property may be desirable as long as this antimicrobial spectrum is restricted to pathogenic microorganisms but it cannot be excluded that it will not affect the normal microbiota of the gut as well. Similarly to other tests, antimicrobial assays may lead to false negatives, i.e., strains that are capable of biosynthesizing antimicrobial(s) but they do not produce it under the *in vitro* conditions employed. In addition, antimicrobials of probiotics are generally perceived as safe and in most cases the toxicity to host’s cells is rarely investigated. There is a clear need for more elaborate assays that would better represent the complex interactions between the probiotics and the host microbiome to understand the consequences of the *in situ* production of antimicrobials by the former.

### IMMUNOMODULATION

Modulation of host immunity is one of the most commonly proposed health benefits attributed to the consumption of probiotics. Probiotic selection that is correlated to the protection against microbial pathogens has been associated with the stimulation of antibody secretion, as well as cell-mediated immune responses (Cross, 2002). Evaluation of the bacterial translocation in the GIT can be used to screen potential probiotic strains, considering that some strains may be capable of triggering dendritic cells (DCs) or M cells from the Peyer’s patches and thereby manage to cross the epithelium (Corthesy et al., 2007). In most of the *in vitro* experiments, researchers have attempted to reconcile the mechanisms underlying the complex and dynamic immune interactions of the gut by using co-culture models (Cencic and Langerholc, 2010) or 3D models (Borchers et al., 2009). The use of three component models (epithelia, immune cells and microbiota) closely mimics the *in vivo* situation (Fontana et al., 2013). In addition, several reports highlight the complex interaction between the host immune system and different bacterial compounds, including chromosomal DNA, cell wall components such as lipoteichoic acids and peptidoglycan, as well as soluble metabolites (Corthesy et al., 2007). In these assays, cytokines like IL-5, IL-10, IL-12b, IL-17a, IFN-γ, TNF-α, and TGF-β, as well as the levels of the secretory immunoglobulin type A (sIgA) are used to assess stimulation of the immune response and the inflammation status (Miettinen et al., 1998; Steinberg et al., 2014).

IBD covers a family of chronic diseases affecting the GIT, having as most common forms Crohn’s disease (CD) and ulcerative colitis (UC). Propitious functions of the probiotics for alleviation of IBD symptoms involve the ability to restore biodiversity within the microbiota, the antagonism against pathogens, the improvement of mucus production, the stimulation of epithelial proliferation, the modulation of intestinal permeability and the mediation of pro-inflammatory effects (Scaldaferri et al., 2013). *In vitro* models for probiotic selection in IBD research are alike to those mentioned previously. Specifically, assays that investigate the antimicrobial activity against microbes that help alter microbial biodiversity within the gut, the regulation of epithelial tight junctions and the induction of an anti-inflammatory cytokine profile (high IL-10/IL-12 ratio) from immune cells, are commonly used (McKay et al., 1997; Foligne et al., 2007).

Allergy is a complex immune response to environmental or food antigenic stimuli and it is mostly correlated with the hypersensitivity reaction mediated by the interaction of immune cells coated with allergen-specific IgE that requires the involvement of T-cells with a Th2-skewed cytokine profile (Furrie, 2005). Th2-skewed immune cells have extensively been studied to select probiotic strains that exert certain immune-stimulating properties for further *in vivo* use (Fujiwara et al., 2004). Alleviation of allergic symptoms has also been correlated to the induction of the immunosuppressive cytokines IL-10 and TGF-β or the reduction of T-cell proliferation (Smits et al., 2005). The β-hexosaminidase release assay helps to identify potential anti-allergic probiotics, since the secretion of this molecule corresponds to a hallmark of allergic reactions resulting from exposure of mast cells to antigens (Kim et al., 2013).

Disturbance in the balance of the normal microbiome in different body niches can lead to inflammation. Probiotics known for their anti-inflammatory cytokine profile from immune cells can be efficacious in moderating this inflammation as in the case of caries and periodontal disease or of functional digestive disorders such as the irritable bowel syndrome (IBS; Krasse et al., 2006; Gill et al., 2009).

While we found the measurement of cytokine levels produced by peripheral blood mononuclear cells (PBMCs) upon stimulation with probiotics to be a reliable and reproducible technique to identify strains with potential pro- or anti-inflammatory properties, the *in vivo* relevance can be questioned as the test involves only one type of immune cells and ignores the complexity of the *in vivo* communication between different cell types. The model is also blind for the differences between the innate and the adaptive immune system. Moreover, the model does not reflect differences of the immune system in relation to certain pathologies. Despite these limitations, experiments with PBMCs are of major interest, as they will allow to quantitatively classify strains and select strains with opposite profiles, e.g., for further mechanistic investigation.

### CARDIOVASCULAR DISEASES (CVDs)

Several reports have demonstrated that manipulating the gut microbiome with probiotics may affect host metabolism and ultimately reduce the risk for CVDs. Increased bacterial BSH activity in probiotic strains can result in decreased body weight gain, lower levels of plasma cholesterol, and liver triglycerides (Cani and Van Hul, 2015). Bile salt hydrolyzing activity can be evaluated qualitatively by the plate assay method using taurodeoxycholic acid (TDCA) sodium salt (Dashkevicz and Feighner, 1989), and quantitatively by high-performance liquid chromatography (Smet et al., 1994). The deconjugation of bile salts can lead to secretion of cholesterol and lipids via the fecal route (Zheng et al., 2013). An *in vitro* assay for the conversion of cholesterol to coprostanol by the action of bacterial cholesterol reductase has also been described (Lye et al., 2010). In relation to cholesterol, coprostanol is less absorbed in the intestine and it can be easier removed with feces. Furthermore, the assimilation of cholesterol present in the growth media by probiotic strains has been tested *in vitro* (Tomaro-Duchesneau et al., 2014).

Probiotic strains and metabolic by-products potentially confer benefits to the heart by the prevention and therapy of heart syndromes, as well as by lowering serum cholesterol (Ebel et al., 2014). The ACE enzyme has a key role in the rennin–angiotensin system which controls the arterial blood pressure and the equilibrium of water and salt in the body. The hydrolysis of angiotensin I to angiotensin II, which is a strong vasoconstrictor agent from the ACE enzyme, can lead to an increase in blood pressure. During proteolysis of extracellular proteins like casein, peptides are being released that may inhibit ACE-I activity which is used as a screening tool (Papadimitriou et al., 2007).

There are several lines of evidence that support the positive implication of probiotics to the prevention of cardiovascular diseases (Ebel et al., 2014). However, the actual mechanisms involved are not well understood and thus the *in vitro* assays available for this type of health claims are relatively restricted. Improving the barrier is generally considered an effective way to decrease basic physiological inflammation of e.g., adipose tissue, contributing to a reduced risk for the development of overweight and metabolic syndrome and therefore positively affecting CVD risks. A general comment can be made regarding the currently available assays for cholesterol absorption or its conversion to coprostanol. The length of these *in vitro* test protocols (often more than 20 h) may not match the *in vivo* time window for the absorption of cholesterol in the intestine after emulsification.

### ANTICANCER

The gut microbiota is considered to be related to the risk of cancer and it has been suggested that consumption of probiotics may decrease this risk. The important role of probiotics in retarding carcinogenesis is attributed to their ability to influence metabolic, immunologic, and protective functions in the body (Wollowski et al., 2001). Antimutagenic activities of probiotics have been evaluated by the Ames test (Burns and Rowland, 2004). Probiotics also exerted an antigenotoxic activity related to decreased DNA damage of colon cells treated with carcinogens as evaluated by the “comet assay” (Pool-Zobel et al., 1996). The nitrosamine degrading assay (Duangjitcharoen et al., 2014) and the evaluation of the antioxidant properties of bacterial samples, i.e., intact cells and cell-free culture supernatants, are used for the detection of potential probiotics against cancer (Amaretti et al., 2013). Bacterial cell-free culture supernatants of probiotic strains have also been tested for preventing colon cancer cell invasion *in vitro* (Choi et al., 2006). Strains showing *in vitro* inhibitory activity on tumor cell proliferation, induction of apoptotic cell death, and ability of cellular sphingolipidic metabolism, have been recognized as promising candidates for cancer prevention (Castro et al., 2010). Moreover, a possible mechanism of anti-carcinogenic properties of probiotic strains involves the physical binding by the bacterial cell of the mutagenic compounds, such as heterocyclic amines (HCAs). Specifically, bacterial strains that are able to sequester HCAs could decrease their absorption by the human intestine via their elimination through feces carryover (Faridnia et al., 2010). Furthermore, *in vitro* removal of toxins and toxic metals present in aqueous solutions or in drinking water has been studied for selected probiotics (Halttunen et al., 2007; Nybom et al., 2008). Also, luminal short chain fatty acids (SCFAs), produced in the colonic lumen during bacterial fermentation, are known anti-carcinogenic agents within the gut (Commane et al., 2005). In the past, the influence of probiotic bacteria on the production of SCFAs by fecal bacteria was studied *in vitro* using batch-culture and continuous-culture techniques (Sakata et al., 2003). *In vitro* GIT models as described above can also be used.

The aforementioned *in vitro* assays for anticancer properties of probiotic bacteria provide minimal information about the actual *in vivo* efficacy. On one hand, probiotic bacteria showing antimutagenic and/or antigenotoxic activities may exert generalized prophylaxis against gut related cancers. On the other hand, probiotics producing SCFAs may have a more direct effect by selectively killing cancer cells as it has been demonstrated for propionic acid bacteria (Cousin et al., 2012) or by assisting in the renewal of the colonic epithelia by butyric acid (Mariadason et al., 1999) or its effect on histone deacytelase (Boffa et al., 1992).

Generally, the existing *in vitro* assays are not sufficient to truly screen probiotics for anticancer properties and thus *in vivo* assays will be necessary. In any case, the idea of using bacteria (some of which are known probiotics, like bifidobacteria) in the treatment of cancer is gaining momentum (Chong, 2014). As the mechanisms of cancer prevention and therapy become clearer, new and more elaborate *in vitro* assays may be developed in the future.

### ADDITIONAL HEALTH BENEFITS

There are several additional health benefits that have been attributed to probiotics. Lactose intolerance, attributed to an insufficient amount of lactase in the small intestine to early hydrolyze lactose, is an important problem when consuming milk or lactose containing foods (Kim and Gilliland, 1983; Céspedes et al., 2013). Dairy products containing probiotic bacteria could aid the digestion of lactose by their β-galactosidase enzyme when crossing or colonizing the gut. Screening for β-galactosidase activity in potential probiotics is assessed through the hydrolysis of the *o*-nitrophenol-β-galactopyranoside (Hughes and Hoover, 1995).

Other nutritional effects of probiotics relate to the production of vitamins. They play a major role in helping humans to meet their needs for these essential nutrients (Eck and Friel, 2013). *In vitro* studies have documented the capacity of some probiotic strains to synthesize vitamin K, folic acid, vitamin B2, and B12 (Pompei et al., 2007).

Probiotics may also have a protective role against oxidative stress in the host (Songisepp et al., 2005). Oxidative stress has many physiological consequences to the host including aging, carcinogenesis, etc. Evaluation of the antioxidative activity of probiotics is usually assessed by the linolenic acid test (LA-test; Kullisaar et al., 2002).

Furthermore, the role of oxalate-degrading bacteria in the treatment of kidney stone disease is of particular interest (Abratt and Reid, 2010). Identification of potential probiotic strains through the evaluation of oxalate degradation by pure cultures has been reported (Campieri et al., 2001).

Lastly, volatile sulfur compounds (VSCs) produced by oral bacteria can cause halitosis. An *in vitro* test was developed by Kang et al. (2006) in which the influence of LAB on VSC production by *Fusobacterium nucleatum* could be assessed. Interestingly, inhibition of VSC production was related to co-aggregation of the LAB strains with *F. nucleatum* as well as the production of H_2_O_2_ by the former.

### FUTURE PERSPECTIVES FOR THE USE OF *IN VITRO* TESTS IN PROBIOTIC RESEARCH

There are several *in vitro* assays that are being employed in an attempt to predetermine or document probiotic properties in relation to health claims. Even though such assays are useful to screen probiotic candidates they exhibit variable effectiveness. Regulatory authorities have attempted the standardization of the *in vitro* assays by publishing detailed protocols and directives. Unfortunately, even a superficial examination of the literature reveals that *in vitro* tests are being performed in a rather arbitrary manner. This makes it difficult to compare findings between different studies. Reproducibility issues have also been reported, making it difficult to rely solely on the outcomes of *in vitro* tests for the selection of probiotic strains. Apparently, *in vivo* assays may be more appropriate but in most cases they cannot be used for high throughput screening due to the increased cost and for ethical reasons. Therefore, *in vitro* screening is and will be an indispensable part of the discovery of new probiotics. More research is needed to improve and standardize the available experimental protocols aiming at improving reproducibility and decreasing the percentages of false positively or negatively identified strains with probiotic potential. Additionally, novel methods might need to be developed that will extend the health-promoting properties currently assessed with *in vitro* tests.

## DISCOVERING PROBIOTIC MICROORGANISMS: *IN VIVO* APPROACHES

### THE RIGHT MODEL FOR THE RIGHT PURPOSE

**FIGURE 1:**
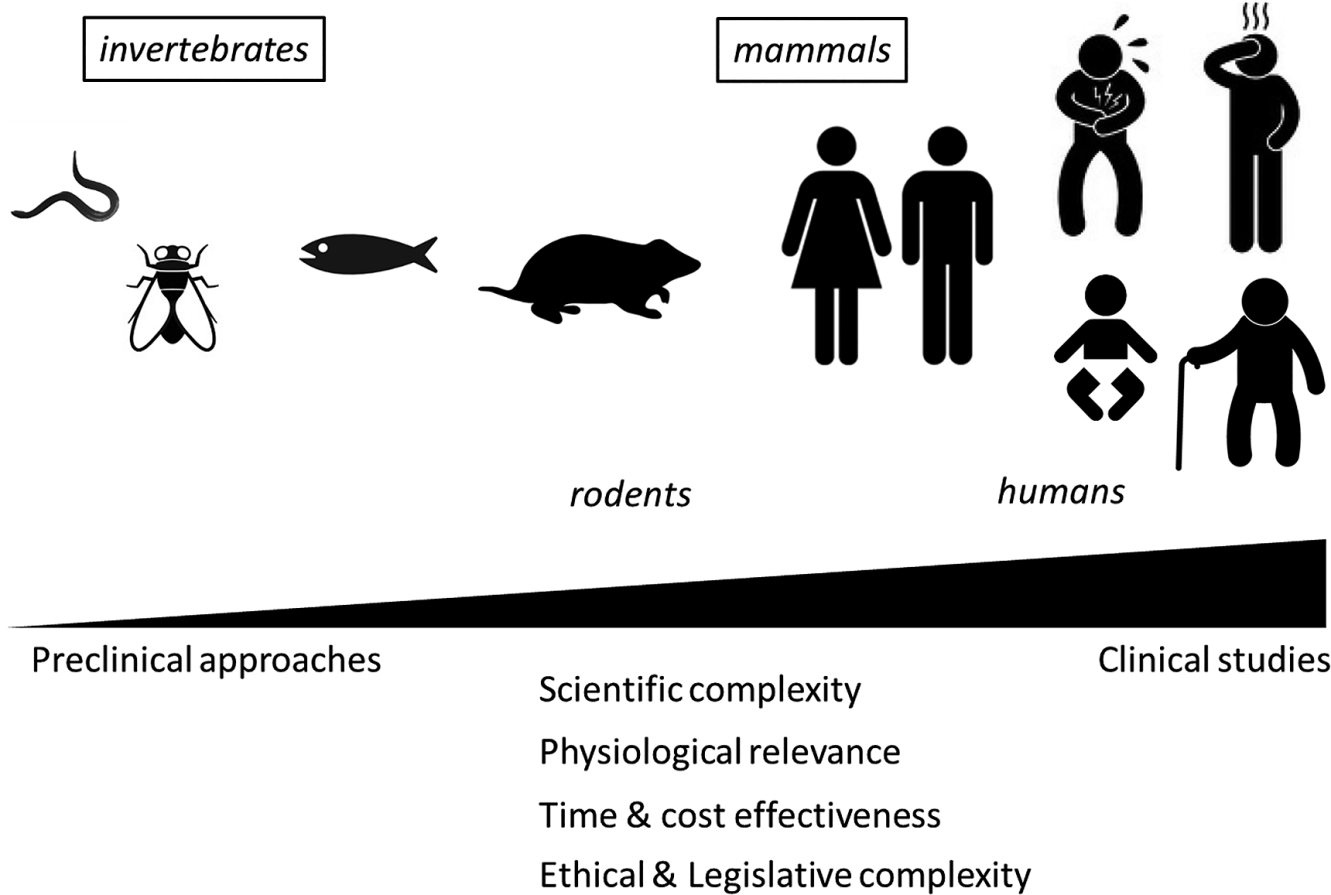
**Progressive complexity of *in vivo* models used to support probiotic health effects**.

The use of *in vivo* approaches to explore probiotic potential resulted in the description of a huge diversity of biological models of various complexity, ranging from simple multicellular organisms such as worms and invertebrates over sophisticated knock-out (KO) models in rodents to human clinical trials in different types of the population (**Figure 1**; **Table 2**). While all these models can teach us something, they also represent certain disadvantages and integrating results from different models remains difficult. Therefore, while final assessment of probiotic functionality should ideally be performed directly in the target population (general population or a subgroup with a given condition; Rijkers et al., 2010), the pre-selection of strains to be included in these expensive clinical trials might need to be made using appropriate *in vivo* models.

**Table 2:** Advantages of using small animals/rodent models for probiotic research support.

Functionality	Possible intervention/improvement	Representative references
Physiological relevance for humans (Immune system, neuroendocrinological system)	Transgenic mice (knock-out/ knock-in)	Helm and Burks (2002)
Closely related innate and adaptive immunity (PRRs and signaling cascades, secretory Ig, T and B cells, DCs, etc.)	Tissue-specific knock-out	Sodhi et al. (2012)
Sharing of similar immune response types (Th1, Th2, Th17, Treg cells and cytokine responses)	Conditional knock-out	Sodhi et al. (2012)
Hosting complex microbiota (gut, vagina, lung, skin)	Humanized mice	Martin et al. (2008)
	Axenic mice	Verdu and Collins (2004)
	Monocolonized mice	Eaton et al. (2011)
	Microbiota transplantation	Le Roy et al. (2013)
	Co-housing	Henao-Mejia et al. (2012)
	Selective antibiotic treatment	Ichinohe et al. (2011), Viaud et al. (2013)
Responsiveness to many infectious, immune and other disorders	Allergy, inflammation (asthma, COPD, IBD, etc.)	Kim et al. (2014)
	Bacteria, virus, fungi and parasites pathogens	Alak et al. (1997), Villena et al. (2011), Collins et al. (2014), Kikuchi et al. (2014)
	Neurologic disorders (EAE, visceral pain)	Eutamene et al. (2007), Rousseaux et al. (2007), Kwon et al. (2013)
	Stress, cognitive functions	Gilbert et al. (2013), Hsiao et al. (2013)

Indeed, while rudimentary models such as *Caenorhabditis elegans*, or *Drosophila* exhibit obvious benefits for (large) screening purposes, they are also not devoid of relevance in deciphering more universal signaling pathways, even related to mammalian innate immunity, as shown by the work of the Nobel prize winner Hoffmann and his team (Vogel, 2012). *C. elegans* was successfully applied to establish the anti-infective, antioxidative and lifespan extending impact of lactobacilli (Wang et al., 2011; Grompone et al., 2012). The prototype worm is currently proposed to screen probiotics (Park et al., 2014), or to establish antitumor activity (Fasseas et al., 2013). In a similar way, the fly is useful to explore metabolic, immune and antioxidant effects of the *Lactobacillus*-host mutualism (Jones et al., 2013; Matos and Leulier, 2014).

Quite recently, the zebrafish (*Danio rerio*) has garnered intense interest as a human disease model (Lieschke and Currie, 2007) due to its many advantages as an experimental vertebrate. It now appears that the zebrafish can be used for high-throughput screening (e.g., of drug libraries) in the discovery process of promising new therapeutics (Lessman, 2011). The latter was successfully developed for probiotics, showing that probiotic administration may enhance the zebrafish welfare by modulating the innate immune response and improving hepatic stress tolerance, involving stress and apoptosis genes, and genes from the innate immune system (Gioacchini et al., 2014; Rieu et al., 2014). Of note, zebrafish can also partly mimic characteristics of IBDs when larvae are subjected to chemicals such as trinitrobenzene sulfonic acid (TNBS; Fleming et al., 2010) or when encountering unfavorable conditions, including dysbiosis of the intestinal microbiota (He et al., 2013). Modifications include colitis susceptibility genes like *NOD1* and *NOD2* (Oehlers et al., 2011), enabling the routine evaluation of anti-inflammatory compounds.

### RODENTS AS THE NECESSARY COMPROMISE

The small animal models mentioned above clearly meet the needs for cost effective and public-acceptable screening but are still far away from an integrated mammalian physiology. Therefore more pertinent experimental models are required for the evaluation of probiotic functionality, allowing to study various dynamic states and when addressing specific diseases with multifactorial origins.

Accuracy of the results of animal models is not always in perfect accordance with human outcomes and may sometimes appear problematic as it was for example recently stated: “Inflammatory findings on species extrapolations: humans are definitely no 70-kg mice” (Leist and Hartung, 2013; Seok et al., 2013). Whether animals can be used to predict human response to drugs, chemicals or foods (including probiotics) is apparently a contentious issue. While some will promote a ban on animal experimentation which lacks scientific evidence for human predictivity (Knight, 2007), the relevance of e.g., mice disease models for humans has been judged rather positively during a dedicated European Commission workshop held in the UK^1^. However, a certain level of criticism on the relevance of animal models does apply. Shanks et al. (2009) illustrate with numerous examples their importance, or lack thereof, in the different steps of the complete research process. They discuss, as an example, the bioavailability differences between primates, rodents, and dogs. When specifically considering probiotic interactions with the host microbiota, a deeper comprehension of the symbiosis between animals and bacteria is key to understanding the complex biology of environmental, genetic and microbiome interactions in relation to human health and disease evolution. Kostic et al. (2013) reviewed how model systems are influencing the current understanding of host–microbiota interactions by exploring recent human microbiome studies. They conclude that experimental model systems, including mice, fish, insects, and the Hawaiian bobtail squid, continue to provide critical insight into how host–microbiota homeostasis is constructed and maintained. Taking also into account the dynamics of the human microbiome through human life stages, predictive value of simple models may indeed have its limits, but their use may be crucial in understanding mechanisms of interactions and regulation of metabolic, physiological, and immunological activities.

Effective use of rodent models will depend on extreme standardization, including the microbiota composition. Relevance for the human situation needs to be considered at any time, as e.g., many bacterial species which are commensal for humans can be pathogenic in mice and *vice versa* (Baker, 1998; Pan et al., 2014). Despite all these possible drawbacks, small animals such as rats and mice will inevitably be continued to be used as models to address numerous research questions related to probiotics, including the evaluation of immune and metabolic responsiveness, regulatory processes or neuro-endocrinological and nutritional aspects, which all play important roles in the complex microbiota–host relationships. Moreover, small animals permit to mimic specific diseases, e.g., by using genetically modified specimens (conditional and tissues-specific knock-in/knock-out mutants, **Table 2**) or specific chemicals (e.g., TNBS to induce intestinal inflammation) and infectious challenges, while manipulation of the microbiota allows to question the role of (a) specific microorganism(s) (**Table 2**).

### FITTING ETHICS AND LEGISLATION

Controversy on animal experimentation has always been high. Researchers in need of animal models have to cope with ethical and legal considerations, as well as with a public opinion that often is not fully aware of the economic and societal importance of the research envisaged, nor of the regulatory context which also limits the unethical use of animal suffering. Researchers are also actively required to ensure that animal models (i) are scientifically (and statistically) validated (ii) cannot be replaced by *in vitro* alternatives and (iii) minimize animal suffering by limiting the number of animals and the length of the experimentation to what is statistically required. Research strategies and methods should be challenged continuously and reviewed objectively with respect to the 3Rs rule established more than 50 years ago, i.e., use opportunities to replace, reduce and refine (Russel and Burch, 1959). In addition to the proper management of pain by analgesia and anesthesia, welfare accommodations improved tremendously following the most recent US and EU animal housing guidelines. Mice and rats should be allowed sufficient space of adequate complexity, allowing to express a wide range of normal behaviors, and providing enrichment possibilities to promote physical exercise, foraging, manipulative and cognitive activities, whenever possible (Richmond, 2000).

In this review we do not focus on the use of large mammals (pigs, dogs, or monkeys) as these are not widely available and ethical considerations are considerable. Non-invasive dietary interventions with pigs, however, may have an interest as the GIT of pigs resembles very well the human GIT.

### MODELING DISEASES TO SUBSTANTIATE HEALTH EFFECTS

Probiotic activity is situated on three main levels (Rijkers et al., 2010): (i) influence on the growth or survival of pathogenic microorganisms in the gut lumen (level 1), (ii) improvement of the mucosal barrier function and the mucosal immune system (level 2) and, (iii) beyond the gut, effects on the systemic immune system, remote organs and systems such as the liver and the brain (level 3). Correspondingly, *in vivo* approaches intended to substantiate probiotic effects might consider these three levels through gastrointestinal infection models, mucosal immune disorder models and models dealing with dedicated experimental neuro-metabolic pathologies respectively.

Maintaining or improving overall “health” is difficult to demonstrate in humans and *a fortiori* in animals. Health claim evidence for probiotics obviously starts by proving innocuousness of the probiotic strain and its matrix and by clearly establishing its safety, using adapted procedures (Vankerckhoven et al., 2008; Sanders et al., 2010). In a second step, the model must fit the purpose of showing convincingly the projected functionality of the probiotic strain, either in a prophylactic or therapeutic way, or demonstrating a reduction of risk activity. This functionality must be illustrated either using read-outs that are relevant for the human situation, in appropriate terms of expected changes in metabolism, physiology, immunology, etc. or by a measurable limitation of the severity or frequency of disease. Discriminating, relevant physio-pathological markers are crucial and should reflect the targeted probiotic functionality. Clearly, the anti-infectious impact of probiotics, depicted for example by the survival rate of a defined pathogen, can only be considered for that particular pathogen and should not be extended to other infections. Consequently, optimal read-outs have to be selected as clear markers of pathology. In the case of inflammation, various pathological as well as beneficial changes may take place. For instance, we routinely determine IL-10 production in inflamed tissues and observed increased amounts of this anti-inflammatory cytokine in the colon of mice following TNBS or dextran sodium sulfate (DSS) treatment, as compared to healthy mice. While IL-10 is a “regenerating” mediator, rapidly induced by injured tissues in response to damage (Barada et al., 2007), it is not an appropriate marker for efficacy in inflamed situations, where IL-10 is to be considered a marker of inflammation. However, in the context of a healthy mucosa, probiotics and other prophylactic anti-inflammatory drugs may be able to substantially increase baseline levels of IL-10 and TGF-α, and thus prevent further injuries.

### INTESTINAL MODELS FOR INFLAMMATION AND INFECTION

For the evaluation of the anti-inflammatory activity of bacteria, several colitis models can currently be used. In many cases the reduction of chemically induced inflammation, e.g., induced by TNBS or DSS, is measured (see Claes et al., 2011 for a complete review). These models are mostly acute models, while chronic ones are less common. They obviously only mimic symptoms of IBD such as UC and CD but do not cause the real disease. Therefore, each model has its own specificity and some interventions may have opposite effects in different models. TNBS evokes an inflammatory process involving T cells while the acute DSS model is more likely to induce epithelial barrier disturbances (Foligne et al., 2010a).

The IL-10 KO mouse model may also be used to study probiotics, but the spontaneous colitis that progressively will occur is not homogenous, difficult to control in time and highly dependent on animal facility conditions. Sometimes additional treatments such as *Helicobacter* infections or small amounts of DSS are required to trigger the onset of inflammation. Clearly, the choice of a model will depend on the putative mode of action of the probiotic used. The IL-10 KO mouse model abolishes a key regulatory cytokine and may thus exclude a virtually protective probiotic if the main mechanism is IL-10 dependent. Similarly, while *nod1*/*nod2* KO mice are good models for CD, the fact that they lack the cellular receptor for peptidoglycan and derived anti-inflammatory products (i.e., certain muramidyl di- or tri-peptides), will at the same time exclude the efficient study of bacteria that execute their anti-inflammatory effect in this way (Macho Fernandez et al., 2011). In general, KO mice models, especially those deficient for key receptors such as TLRs, are appropriate for case-by-case studies, often aiming at confirming specific mechanisms of action, but are less suitable for broader screening purposes.

Finally, some other models of colitis involve adoptive transfer of specific T cells (CD4^+^CD45rb) in immune-deficient animals (SCID, Rag^-/-^) allowing to explore the impact of probiotics on adaptive immunity. *Citrobacter rodentium* is a murine pathogen that induces, depending on the genetic background of the mice, variable degrees of infectious and inflammatory responses, ranging from infectious colitis to severe and fast lethal inflammation. It shares several pathogenic mechanisms with enteropathogenic *E. coli* (EPEC) and enterohaemorrhagic *E. coli* (EHEC), two clinically important human gastrointestinal pathogens, showing severe adhesion-based virulence in the intestinal mucosa. The models also display crypt hyperplasia, demonstrating similarities with pre-carcinogenic states. Some probiotics have been shown to positively alleviate *C. rodentium*-induced colitis as demonstrated for lactobacilli, bifidobacteria, propionibacteria, yeasts and spores of *Bacillus subtillis* (Chen et al., 2005; Johnson-Henry et al., 2005; Wu et al., 2008; Foligne et al., 2010b; Collins et al., 2014). This model, when appropriately used, can be considered a model of choice to investigate overall probiotic functionality (Borenshtein et al., 2008) as it reveals information on the anti-infectious as well as the anti-inflammatory potential of the strains tested.

*Clostridium difficile* can be the causative agent of primary and recurrent antibiotic-associated diarrhea and colitis in hospitalized patients. Mice models to mimic this type of infection exist but require the administration of a broad range of antibiotics that are not always compatible with the planned probiotic interventions (Chen et al., 2008; Sun et al., 2011). Therefore, the preferential use of yeasts such *Saccharomyces boulardii* has been suggested (Barc et al., 2008), or the window of intervention is to be kept quite short to demonstrate substantial effects (Kaur et al., 2010). Partial efficacy has been shown on inflammation or stool consistency parameters, although the infection was not completely cured (Fitzpatrick et al., 2011). Further efforts are needed to optimize the model for more detailed study of promising probiotic strains.

Similarly to *Clostridium difficile*, a *Salmonella typhimurium* infection in mice requires the administration of antibiotics during the colonization (Hapfelmeier and Hardt, 2005), limiting also the readouts of the model. However, infectious challenges with *S. enterica* can be useful to study mortality and translocation (Zoumpopoulou et al., 2008; de LeBlanc Ade et al., 2010; Zacarias et al., 2014), focusing on indirect anti-inflammatory effects and on the impact of the probiotics on the intestinal barrier.

Finally, listeriosis is not spontaneously modeled in mice and infectivity would require genetically modified humanized animals having the necessary receptor to allow the pathogen to attach and disseminate within the host. When established, it might allow to explore anti-infective probiotic potentials against *Listeria* (Archambaud et al., 2012).

### CANCER MODELS

While animal models exist for chemopreventive and chemotherapeutical drugs for e.g., mammary and ovary cancer, bladder, and prostate cancer, esophagus and colon cancer, lung and pancreas cancer, skin head and neck cancer, most studies involving probiotics focused on colorectal cancer (CRC), as it represents the most common malignancy of the GIT and has been linked to dietary habits and a Western lifestyle. Many indications have indeed pointed toward the importance of the microbiota in the increased risk for CRC development. Several possible mechanisms for reducing the risk for CRC development have been suggested, each supported by *in vitro* and *in vivo* models (Uccello et al., 2012). Probiotic intervention intends to alter the metabolism of the microbiota by reducing intestinal enzymes that promote the production of potential carcinogenic substances: β-glucuronidase that produces aglycons, or nitroreductase and azoreductase, which produce free amines from aromatic nitroso compounds and azo dyes respectively (Goldin and Gorbach, 1984).

A second mechanism includes the direct inactivation of potential carcinogenic compounds, such as mutagenic derivatives of heterocyclic aromatic amines. Commensal bacteria have been found to bind or metabolize these compounds, resulting in reduced mutagenicity in HCA exposure models (Kumar et al., 2010), reduced bioavailability of other mutagens in the GIT and tissues in mice (Orrhage et al., 2002) or increased detoxification in rats (Challa et al., 1997). Another way to study probiotic efficiency in reducing the prevalence of CRC is the IL-10 KO mice model (O’Mahony et al., 2001), leading to spontaneous colitis and colon cancer development.

Improving the host’s immune response by activation of antigen-presenting cells, natural killer cells or subsets of T and B cells is another way to promote antitumor activity in mice (Sekine et al., 1985) and may explain some of the observations in syngeneic mice and guinea pigs on Lewis lung carcinoma and line-10 hepatoma (Matsuzaki et al., 1985), as well as in bladder cancer in man (Aso et al., 1995). The same probiotic strain was also shown to have a positive effect on transplantable tumor cells and to suppress chemically-induced carcinogenesis in rodents (Matsuzaki et al., 1988). A component of the polysaccharide peptidoglycan complex was shown to improve colitis-associated cancer in mice (Matsumoto et al., 2009). Measuring survival rates of mice injected with tumor cells is another model that can be used to test or compare the potential of different probiotic strains to increase cellular immunity (Lee et al., 2004).

Anti-proliferative effects by regulation of apoptosis and cell differentiation have been described in response to the carcinogen azoxymethane (AOM; Le Leu et al., 2005), which may be linked to reduced levels of ornithine decarboxylase involved in polyamine biosynthesis (Moorehead et al., 1987). Butyrate also enhances cellular differentiation and reduces proliferation (Topping and Clifton, 2001). Butyrate may be used as an energy source by the colonocytes and together with other SCFAs they may reduce the colonic pH and the concentration of secondary bile salts. In addition, conjugated linoleic acids (CLAs) were also shown to have anti-inflammatory and anti-carcinogenic effects (Kim and Park, 2003; Ewaschuk et al., 2006; Evans et al., 2010), possibly through the activation of PPARγ.

Besides for CRC, the Apc^Min^ mouse model can also be used in the case of mammary tumorigenesis (Moser et al., 1995). Chen et al. (2009) used this model to examine the quantitative and mechanistic effects of probiotic yeast on the induction of intestinal tumors. Clearly a large number of models exist for the investigation of probiotic activities on cancer, reflecting the many possible mechanisms of probiotic activity, as well as the intense cancer research activity.

### MODELS LOOKING INTO METABOLIC DISORDERS

Metabolic syndrome (MS) is a complex multifactorial disorder involving genetic predisposition, life style, diet, and environmental factors and is almost always accompanied by an increased risk of cardiovascular diseases and type 2 diabetes, and possibly also non-alcoholic fatty liver disease (NAFLD) and hypertension. Obesity is the most important precursor for MS, especially on a longer term. Pro- and prebiotics can play a role in weight management, as obesity was found to be linked to a dysbalance of the microbiota, both in mice and man (Ley et al., 2005). Turnbaugh et al. (2008) investigated the inter-relationship between diet, microbial ecology of the gut and energy balance using a Western diet-induced obesity model in mice. This diet induced a dominance of the Firmicutes in the distal gut microbiota which could be reversed by subsequent dietary manipulations to limit weight gain. Interestingly the transplantation to germ-free lean mice of the microbiota from mice with diet-induced obesity, increased significantly more the adiposity in the recipient mice than transplants from lean donor mice. Possible mechanisms include a change in intestinal permeability, leading to decreased translocation of lipopolysaccharides in the microbiota of lean mice and therefore to decreased inflammation and abdominal adiposity (Cani et al., 2008). Also the importance of *A. muciniphila* in this process has recently been illustrated (Everard et al., 2013). Overall these observations have fuelled the idea of using probiotics and prebiotics in dietary strategies for weight management (Nicholson et al., 2005; Cani et al., 2009). Again, several mechanisms, and thus several models, can be considered to improve gut microbial balance: CLA production (Lee et al., 2006), polyphenol supplementation, low amounts of probiotics (Rastmanesh, 2011), prebiotic intake, decreased food intake, increased satiety, increased barrier function or ways to decreased abdominal adiposity or total cholesterol levels (Yadav et al., 2008). Since it is impossible to describe all MS related animal models here, it is important to note that the cause/consequence relationship is not clear when only considering the composition change of the microbiota related to obesity, as many of the models involve high-fat diets, also directly affecting the microbial composition (Hildebrandt et al., 2009). The use of germ-free mice with standardized nutrition might help to study the direct impact of a particular microbiota composition on MS. The observed shifts in the microbiota composition may have an impact on the barrier function, but they have also been linked to functional shifts (e.g., production of SCFAs) in the microbiota which can contribute to an obese phenotype (Turnbaugh et al., 2006). Important to notice as well, is the observation that the situation in humans seems to be different from mice, since bacteroidetes-related taxa were either reported to increase, to remain unchanged, or to decrease after weight loss (Duncan et al., 2008; Nadal et al., 2009). The detection of the *ob* and *db* genes in mice led to the development of several animal models to study obesity effects (Ingalls et al., 1950; Hummel et al., 1966; Comuzzie and Allison, 1998). The *ob* gene, located on chromosome 6, encodes the hormone leptin and renders *ob/ob* mice massively obese, with marked hyperphagia and mild transient diabetes, while the *db* gene, located on chromosome 4, codes for the leptin receptor and renders the *db/db* mice markedly obese with hyperphagia, but with severe, life-shortening diabetes.

One can conclude that the complex interplay between genetics, environment, diet, the microbiota and its metabolism or the barrier and immune function of the host, make it difficult to develop all-round animal models. Partial mechanisms may need to be put forward for independent evaluation, with the total picture being obtained through the combination of different *in vitro* and *in vivo* models. Translation from animal to man may also prove to be difficult, because of this complexity.

### MODELS FOR AUTO-IMMUNE DISEASES

Similarly to MS, auto-immune disease (AID) covers a broad range of possible diseases (type 1 diabetes, multiple sclerosis, rheumatoid arthritis, pemphigus vulgaris, scleroderma, Sjögren’s syndrome, etc.) involving, besides genetic factors, also an aberrant intestinal microbiota, a disturbed mucosal barrier and altered intestinal immune responsiveness. All these factors share functional links and will therefore determine the models to be considered for more in depth study of probiotic mechanisms or for comparing different strains. Since probiotics have the potential to interfere with all three factors, one will need to try and find strains that have the capacity to change in a positive direction any, or any combination, of these factors. As for the intestinal immune responsiveness, interest will be in anti-inflammatory probiotics, as chronic inflammation underlies many AIDs and is often at the start of its development, as in rheumatoid arthritis. It is also not clear if the AID is caused by inflammation, or the other way around.

Animal models such as the non-obese diabetic mice will spontaneously develop diabetes resembling human insulin-dependent diabetes mellitus (Gepts and Lecompte, 1981; Kataoka et al., 1983). The fact that in these mice a progressive lymphocytic infiltration of autoreactive CD8^+^ T cells into the islets of Langerhans will cause insulitis positioned the model as a model of AID. In another model, considered a good model of rheumatoid arthritis in humans, a probiotic strain prevented the onset of type II collagen-induced arthritis in DBA/1 mice (Kato et al., 1998). Results suggest that the probiotic was able to modify the systemic humoral and cellular immunity and could elicit alterations of the immune state in this model. In general there are many conflicting data on the effect of probiotics in AID. In part this uncertainty comes from the diversity of the strains evaluated, while the different genetic backgrounds of the host might also be an important reason.

### FUTURE PERSPECTIVES FOR THE USE OF *IN VIVO* TESTS IN PROBIOTIC RESEARCH

To increase the accuracy of animal models, multi-humanized mice can be considered, carrying functional human genes, cells, tissues, or organs. Immune-deficient mice are often used as recipients for human cells or tissues, because they can relatively easily accept heterologous cells due to lack of host immunity. Traditionally, nude mice and severe combined immunodeficiency (SCID) mice have been used for this purpose, but many other models have been shown to engraft human cells and tissues even more efficiently (Ito et al., 2008). These humanized mouse models may assist to model the human immune system in various scenarios of health and disease, and may enable the evaluation of therapeutic candidates in an *in vivo* setting more close to human physiology. While those specific humanized mice are commonly used in biological and medical research for human therapeutics, they do not frequently appear in probiotic research yet.

Given the importance of the microbiota for many immune and metabolic functionalities of the host, the development and use of mice models with an artificially composed microbiota, e.g., a human microbiota, might help to better mimic the human condition. The use of axenic or monoxenic mice may help to unravel the “egg or the chicken” question mentioned above. The impact of a dietary intervention with or without a microbiota can learn interesting things about the direct influence of the administered probiotic versus e.g., an indirect metabolic or microbiological effect or can show the direct impact on the immune system of any planned intervention.

As for the ethical problem of using animal models, interesting developments such as seen at the Wageningen University in The Netherlands, may bring some solution in the future. *In silico* solutions may try to represent the interactions of the pig gut, the nutrients and other feed/food components with the residing microbes and with epithelial cells. All elements are considered as nodes in a mathematical model, together with their mutual, quantitative dependencies^2^. Using these model interactions, a number of higher level processes related to intestinal immunity, tolerance and barrier functions can possibly be simulated, and conditions as gut homeostasis could possibly be better understood. On a longer term, the model may even evolve into a dynamic and predictive one, allowing to test hypotheses.

## DISCOVERING PROBIOTICS WITH GENETICS AND OMICS

### STRESS RESPONSES

Over the past two decades several attempts have been made to identify molecular markers that would facilitate the rapid selection of probiotic strains (**Table 3**). To elucidate the complex adaptation mechanisms of probiotic microorganisms under stress conditions, several genetic and omics studies have been conducted in an attempt to identify gene expression and/or protein production patterns related to stress. The exposure of cells to gastric acidity causes reduction of intracellular pH, which adversely affects numerous cell wall and transmembrane-based processes and damages proteins, nucleic acids and other cellular macromolecules. To resist acid stress, microorganisms employ mechanisms aiming at the maintenance of the intracellular pH homeostasis, the repair of macromolecular structures like the cell envelope or ribosomes and other damaged molecules (Lebeer et al., 2008). Heat shock proteins are molecular chaperones involved in the repair of acid-damaged proteins. Many studies have demonstrated that several heat shock proteins, e.g., DnaK, GroES, GroEL, GrpE are induced by acid stress, but their induction varies among different species/strains (Hamon et al., 2014). In parallel, the Clp proteases (e.g., ClpP, ClpE, ClpL) are also induced under acid stress targeting denatured proteins to re-fold them to the appropriate structure or to degrade them if they are beyond repair (Ferreira et al., 2013; Hamon et al., 2014). Genes implicated in DNA repair were also found to be up-regulated under acidic stress (e.g., *uvrB*, *uvrD1, vsr;* Jin et al., 2012). Furthermore, an essential component in the response against low pH is the up-regulation of the F_1_F_0_-ATPase (Sanchez et al., 2008; Jin et al., 2012; Koponen et al., 2012). The various subunits of this multimeric enzyme are being encoded by eight genes found on the *atp* acid inducible operon. The F_1_F_0_-ATPase lowers the cytoplasmic concentration of protons by virtually extruding them to the extracellular environment at the expense of ATP which can ultimately lead to energy depletion and growth arrest. The composition of the cell envelope is also altered upon exposure to acidic conditions to decrease its permeability to protons. Genes and proteins involved in peptidoglycan biosynthesis (e.g., *manB*, *glmU*, *dapA*, glycosyltransferases), in D-alanylation of lipoteichoic acids (e.g., *dlt* operon), in fatty acid (e.g., *fab* genes) and exopolysaccharide (EPS; e.g., Etk-like tyrosine kinase) biosynthesis were induced to ameliorate the cells’ resistance to acid stress (Perea Velez et al., 2007; Jin et al., 2012; Koponen et al., 2012). In addition proteins involved in cell envelope biogenesis (e.g., FabF, RfbB, RfbC) were shown to have a strain-specific role in acid tolerance (Hamon et al., 2014). The gene *luxS* exhibited enhanced expression under acidic conditions in several probiotics, implicating quorum sensing (QS) in acid stress resistance (Moslehi-Jenabian et al., 2009; Koponen et al., 2012).

**Table 3:** Potential gene/protein markers related to different probiotic properties identified during genetic and omics studies.

Gene/protein	Function	Representative references
**Stress responses (acid and bile)**
Heat shock proteins (e.g., DnaK, GroES, GroEL, GrpE)	Repair of damaged proteins	Wu et al. (2010), Ruiz et al. (2012), Hamon et al. (2014)
Clp proteases (e.g., ClpP, ClpE, ClpL)	Refolding or degrading denatured proteins	Ferreira et al. (2013), Hamon et al. (2014)
*uvrB*, *uvrD1, vsr*, helicases	DNA repair	Jin et al. (2012), An et al. (2014)
F_1_F_0_-ATPase	Decrease of intracellular pH	Sanchez et al. (2008), Hamon et al. (2011), Jin et al. (2012), Koponen et al. (2012)
*manB, glmU, dapA*, glycosyltransferases	Peptidoglycan biosynthesis	Jin et al. (2012)
*dlt* operon	D-alanylation of lipoteichoic acids	Perea Velez et al. (2007), Koskenniemi et al. (2011)
*fab* genes	Fatty acid biosynthesis	Koponen et al. (2012), An et al. (2014)
Etk-like tyrosine kinase, *welG*	Exopolysaccharide biosynthesis	Koskenniemi et al. (2011), Jin et al. (2012)
FabF, RfbB, RfbC	Cell envelope biogenesis	Hamon et al. (2014)
*luxS*	Cell-to-cell communication	Moslehi-Jenabian et al. (2009), Wu et al. (2010), Koponen et al. (2012)
*bsh*	Deconjugation of bile salts	Hamon et al. (2011), Koskenniemi et al. (2011), An et al. (2014)
Transporters	Bile eﬄux	Price et al. (2006), Gueimonde et al. (2009), Koskenniemi et al. (2011), Ruiz et al. (2012), An et al. (2014)
**Adhesion**
Mub	Cell-surface proteins with cell wall anchoring motif (LPXTG)	Azcarate-Peril et al. (2008), Mackenzie et al. (2010), Weiss and Jespersen (2010), Turpin et al. (2012)
*slpA*	S-layer protein	Falentin et al. (2010), Ashida et al. (2011), Beganovic et al. (2011), Turpin et al. (2012)
Apf	Aggregation promoting factor	Ventura et al. (2002), Turpin et al. (2012)
*srtA*	Sortase-dependent surface protein	Munoz-Provencio et al. (2012)
*spaCBA*, *spaFED*, *pil2*, *pil3, fim1, fim2, fim3*	Sortase-dependent biosynthesis of pili	Kankainen et al. (2009), Foroni et al. (2011), Gilad et al. (2011), Westermann et al. (2012), Douillard et al. (2013), Turroni et al. (2013)
FbpA, E1 β-subunit of the pyruvate dehydrogenase complex	Fibronectin binding protein	Azcarate-Peril et al. (2008), Weiss and Jespersen (2010), Vastano et al. (2014)
*tad*	Assembly of tide adherence pilus	Westermann et al. (2012)
**Degradation of HMOs and Mucus**
43 kbp gene cluster	Catabolism of HMOs	Sela et al. (2008)
F1SBPs	Import of oligosaccharides	Sela et al. (2008)
β-Galactosidases	Degradations of type-1 and type-2 HMOs	Yoshida et al. (2012)
Glycosylases	Degradation of HMOs	Kim et al. (2009)
Glycosyl hydrolases, exo-α-sialidases, fucosidases, PTS systems, ABC-type carriers, specific permeases, *engBF*, *afcA,* NagBb, AgnB	Mucin degradation	Yang et al. (2007), Ruas-Madiedo et al. (2008), Turroni et al. (2010a, 2011), Kiyohara et al. (2012), Shimada et al. (2014)
*adh*	Adhesion and stimulation of mucin secretion	Mack et al. (2003)
Soluble protein p40	Stimulation of mucin production	Wang et al. (2014)
**Modulation of the immune system**
p40 and p75 proteins and homologues	Activation Akt, promotion of cell growth, inhibition of TNF-α	Yan et al. (2007), Bauerl et al. (2010)
ClpB, Rpf	Potential immunogenic proteins	Gilad et al. (2011)
SLPs (SlpA, InlA, LspA, SlpE, SlpB), additional disperse genetic loci	Regulation of anti- or pro-inflammatory immune responses (e.g., induction of the IL-10 and IL-6 regulatory cytokines)	Konstantinov et al. (2008), Meijerink et al. (2010), van Hemert et al. (2010), Beganovic et al. (2011), Le Marechal et al. (2014)
*ser*	Inhibition of elastases	Turroni et al. (2010b)
Cell surface-associated EPS	Adaptive immune response and protection against the gut pathogen *Citrobacter rodentium*	Fanning et al. (2012)
Flagellin	Induction of human β-defensin 2 (hBD-2)	Schlee et al. (2007)
**Production of antimicrobial compounds**
Bacteriocins	Protection against enteropathogens	Corr et al. (2007)
Genes involved in plantaricin biosynthesis and secretion	Regulation of pro- and anti-inflammatory cytokines of DCs	Meijerink et al. (2010)
**Quorum sensing**
*luxS*	Induction of anti-inflammatory cytokines Adhesion and competitive exclusion of pathogens	Jacobi et al. (2012) Buck et al. (2009), Moslehi-Jenabian et al. (2011)
*lamBDCA* operon, *lamKR* operon	*agr*-like quorum sensing systems	Sturme et al. (2005), Fujii et al. (2008)
Quorum sensing system related peptide (CHWPR)	Induction of *c-myc* and *IL-6* genes in somatic cells	Mitsuma et al. (2008)
**Production of nutrients and other beneficial processes**
Vitamins, essential amino acids, SCFAs	*In situ* production of important nutrients	Falentin et al. (2010), Saulnier et al. (2011)
*fos*	Processing of health-promoting fructooligosaccharides	Ryan et al. (2005), Kim et al. (2009)
ABC carbohydrate transporters	High production of acetate and protection from enteropathogenic infection	Fukuda et al. (2012)
*ccpA*	Influencing blood cholesterol	Lee et al. (2010)

As discussed above, having survived the hostile environment of the stomach, probiotics next have to face bile in the duodenum. Many transcriptomics and proteomics studies have been performed to determine bile resistance factors in probiotic strains. Interestingly, several of the pathways that are activated during acid stress in the stomach seem to be involved in the ability of probiotics to adapt to bile, as well. Responses against bile include the increased expression of molecular chaperones (e.g., GroEL, GroES, HSP20, DnaK), proteases (e.g., Clps, DegQ), DNA repair proteins (e.g., helicase) and of the F_1_F_0_-ATPase (Wu et al., 2010; Hamon et al., 2011; Ruiz et al., 2012; Ferreira et al., 2013; An et al., 2014). Genes involved in EPS (e.g., *eps*, *welG*) or fatty acid biosynthesis (e.g., *acc*, *fab*) have been found down-regulated (Koskenniemi et al., 2011; An et al., 2014). In contrast the *dlt* operon was up-regulated (Koskenniemi et al., 2011). It is generally accepted that cells attempt to protect the integrity of the cell envelope by appropriately regulating cell wall and cell membrane processes (Lebeer et al., 2008). Apart from these generic mechanisms that may also be induced by other stresses, there are some that are obviously specific for bile stress. The BSHs encoding genes were found to be up-regulated under bile stress in many studies (Hamon et al., 2011; Koskenniemi et al., 2011; An et al., 2014). However, this is not always the case indicating differences in the regulation of BSH among probiotic strains (Sanchez et al., 2007). Bile export from the cell is another mechanism to counterbalance bile toxicity. Permeases of the major facilitator superfamily (MFS) have been found to be up-regulated and could play a similar role with the bile-inducible eﬄux transporter BetA. Also ABC (ATP-binding cassette) transporters could play a role in bile expulsion (Koskenniemi et al., 2011; Ruiz et al., 2012; An et al., 2014). Probiotic microorganisms also use multidrug resistance (MDR) bile eﬄux transporters to actively pump bile salts out of the cytoplasm. Several MDR genes (e.g., *betA*, *ctr*) have been reported to be induced under bile stress (Price et al., 2006; Gueimonde et al., 2009).

The molecular basis of the stress physiology of LAB and other probiotics has advanced rapidly over the past years (Bron et al., 2011; Upadrasta et al., 2011). As our understanding of the stress response mechanisms have increased, a plethora of genes could have been selected as molecular markers for identifying robust probiotic strains. However, this approach has not been followed yet for a number of reasons. A closer look at the lists of genes involved in stress resistance reveals that many of them are well conserved and thus their presence does not reveal anything for the strain under investigation (e.g., heat shock proteins, F_1_F_0_-ATPase, etc.). In fact, several of them are housekeeping genes involved in central cellular processes and thus it is unlikely that they will be missing from the bacterial genome. In such cases it is the enzymatic activity of the relevant protein or protein complex that is decisive and it needs to be experimentally confirmed. For example a bile-resistant mutant of *Bifidobacterium lactis* subsp. *animalis* overexpessing the F_1_F_0_-ATPase resisted acid stress better than the parental strain (Sanchez et al., 2006). Finally, at this stage, very few genes could be directly related to the robustness to a specific stress. The presence of BSHs genes as an example, is indicative for resistance to bile stress. However, this information on its own is not sufficient to provide us with the overall behavior of a strain under the multitude of probiotic stresses. The identification of more sequences, linked to their respective specific phenotypes, may lead to the construction of databases with a more predictive value.

### ADHESION TO THE HOST

As discussed above, adhesion to the host’s cells could be a significant characteristic of probiotics. Temporary colonization may be necessary for the probiotic to exert its properties e.g., the competitive exclusion of pathogens and the modulation of the immune system. A potential mechanism for adhesion to the host implicates the binding of molecules exposed on the surface of microbial cells to the mucus layer of the host’s intestine. Mucus-binding proteins (Mubs) hold an important role in the process of probiotics’ adherence to the host. Mubs are cell-surface proteins, characterized by the presence of a *C*-terminal cell wall anchoring motif (LPXTG) and multiple Mub repeats, homologous to the MucBP domains (Boekhorst et al., 2006) which bind to mucins and glycans. Several *mub* genes and Mub proteins have been determined in probiotic strains (Azcarate-Peril et al., 2008; Mackenzie et al., 2010; Weiss and Jespersen, 2010; Turpin et al., 2012). Surface (S-) layer proteins also play a pivotal role in the adhesion of probiotics. The *slpA* gene encoding the surface-layer protein A (SlpA) has been shown to be involved strongly in adhesion capacity (Ashida et al., 2011; Beganovic et al., 2011; Turpin et al., 2012) and has been also identified in the genome of probiotic propionibacteria (Falentin et al., 2010). Some probiotic strains which are devoid of S-layer proteins, encode the aggregation promoting factor (Apf) which shares several features with the S-layer proteins (Ventura et al., 2002; Turpin et al., 2012). Additionally, sortase-dependent surface proteins may also play an important role concerning the adhesion to the host. It has been demonstrated that disruption of the housekeeping sortase gene *srtA* led to reduced bacterial adhesion to epithelial cells (Munoz-Provencio et al., 2012). A major target for bacterial adhesins is fibronectin, an extracellular matrix glycoprotein. A transcriptomics study revealed that the gene encoding a fibronectin-binding protein was significantly up-regulated during incubation in duodenal juice and bile (Weiss and Jespersen, 2010). Genome analysis of a probiotic strain showed that the presence of FbpA protein may be responsible for adhesion to the extracellular matrix of epithelial cells (Azcarate-Peril et al., 2008). In a recent study, Vastano et al. (2014) demonstrated that the moonlighting protein E1 β-subunit of the pyruvate dehydrogenase complex, which is encoded by the *pdhB* gene, is an element related to fibronectin-binding. Some probiotic strains are equipped with proteinaceous surface appendages, such as pili or fimbriae, which facilitate their adhesion to human gut cells. In Gram positive bacteria the assembly of pili relies mostly upon a sortase-dependent mechanism (Mandlik et al., 2008). Gene clusters responsible for the biosynthesis of pili have been identified in the genomes of probiotic lactobacilli (e.g., *spaCBA*, *spaFED*; Kankainen et al., 2009; Douillard et al., 2013) and bifidobacteria (e.g., *pil2*, *pil3, fim1, fim2, fim3*; Gilad et al., 2011; Westermann et al., 2012; Turroni et al., 2013). Moreover, genetic analysis of several probiotic bifidobacteria strains revealed the existence of pilus gene clusters in their genome. Each cluster was organized in an operon and contained the major pilin subunit-encoding gene (*fimA* or *fimP*) along with one or two minor pilin subunit-encoding genes (*fimB* and/or *fimQ*) and a gene encoding a sortase enzyme (*strA*; Foroni et al., 2011). Genes involved in the assembly of the tide adherence (Tad) pilus found in bifidobacteria has also been reported (Westermann et al., 2012). Proteomic and genomic analyses have shown that moonlighting proteins (e.g., ENO, GAPDH, EF-Tu) and proteins related to stress response (e.g., DnaK, GrpE, GroEL, GroES) promote the adhesion of probiotics (Sanchez et al., 2005; Candela et al., 2009; Izquierdo et al., 2009; Gilad et al., 2011; Turpin et al., 2012; Le Marechal et al., 2014).

In contrast to stress related genes, genes involved in adhesion may be more informative about the properties of a strain. The presence of genes encoding adhesive molecules is still considered beneficial for selecting a probiotic strain as it might increase its interaction with the host. The adhesion biology of bacteria (both probiotics and pathogens) is a field evolving fast and detailed molecular mechanisms are being elucidated. These developments are expected to significantly improve the selection of probiotic strains. For example *in silico* analysis of these molecules may help to determine the nature of the adhesion sites (e.g., binding to fibronectin, mucin, etc.). Validation of the predicted adhesive potential of a strain is relatively straightforward with *in vitro* and *in vivo* assays as described earlier.

### HUMAN MILK OLIGOSACCHARIDES AND MUCUS DEGRADATION

Mucins can modulate bacterial colonization as a direct energy source (Derrien et al., 2010). The gene pool of bifidobacteria contains several genes involved in the metabolism of human milk oligosaccharides (HMOs) and host-derived carbohydrates, like mucins. These genes allow the adaptation of these bacteria to the human GIT (Pokusaeva et al., 2011; De Bruyn et al., 2013). Genomic analysis of several bifidobacterial strains has given useful insights about the molecular mechanisms supporting these processes.

The genome sequence of *B. longum* subsp. *infantis* ATCC 15697 was found to contain a novel 43 kbp gene cluster encoding genes predicted to be involved in the catabolism of HMOs (Sela et al., 2008) as well as a great number of solute binding proteins (F1SBPs). The latter are part of ABC transporters and they are associated with the import of oligosaccharides. Furthermore, the expression of specific binding proteins related to HMO isomers import was induced under growth on HMO (Garrido et al., 2011). It was also shown that this probiotic bacterium uses two different β-galactosidases for the degradations of type-1 and type-2 HMOs (Yoshida et al., 2012; De Bruyn et al., 2013). Furthermore, in the genome sequence of *B. animalis* subsp. *lactis* AD011, several glycosylases were identified which are associated with the degradation of HMOs (Kim et al., 2009). Proteomic analysis of *B. bifidum* PRL2010 showed that among the mucin induced proteins were a variety of glycosyl hydrolases, while transcriptional profiling led to the identification of several mucin-induced genes encoding for different components (e.g., exo-α-sialidases, fucosidases, PTS systems, ABC-type carriers, specific permeases; Turroni et al., 2010a, 2011). Two *B. bifidum* strains containing *engBF* and *afcA* genes, encoding for endo-α-*N*-acetylgalactosaminidase and 1,2-α-L-fucosidase respectively, were able to degrade high-molecular weight porcine mucin *in vitro*. The expression of both genes was highly induced in the presence of mucin (Ruas-Madiedo et al., 2008). Moreover, two novel α-*N*-acetylgalactosaminidases from *B. bifidum* JCM 1254 have been identified, NagBb (Kiyohara et al., 2012) and AgnB (Shimada et al., 2014). These enzymes exhibit activity against the core structures in mucin O-glycans. Concerning the probiotic lactobacilli, proteomic analysis of *Lactobacillus fermentum* I5007 after exposure to jejunal environment *in vivo*, disclosed the induction of a glycoside hydrolase, a mucin degrading enzyme (Yang et al., 2007).

In addition, mucins play a crucial role in the protection of the intestinal barrier function. Mucin degradation by intestinal bacteria and its use as a carbon source stimulate goblet cells to increase mucus production. Probiotics may also influence the production of mucin directly during adhesion or through other mechanisms. A mutant of the probiotic *L. plantarum* 299v strain lacking the adhesion gene (*adh*), lost the potential to induce mucin secretion (Mack et al., 2003). A recent study demonstrated that the soluble protein p40 from the probiotic strain *L. rhamnosus* GG, stimulates the activation of epidermal growth factor receptor (EGFR), which promotes the up-regulation of mucin production in goblet cells. Therefore p40 may contribute to the protective mechanism of the intestinal epithelium from injury and inflammation (Wang et al., 2014).

The ability of probiotics to degrade hosts glycans and use host oligosaccharides as an energy source is a very important property. The presence of genes whose products are involved in these processes in the genome of a strain is a clear indication about the adaptation of this particular strain to the GIT. Such strains may have a competitive advantage over other strains in prevailing and colonizing the GIT. The stimulation of mucin production by probiotics is also very interesting as it can facilitate increased binding sites for probiotics and improved gut barrier functions.

### MODULATION OF THE IMMUNE SYSTEM

Probiotic bacteria can modulate the response of the host’s immune system, interacting with IECs and DCs. It was demonstrated that the p40 and p75 proteins, purified from *L. rhamnosus* GG, stimulate activation of protein kinase B (Akt), promote cell growth, and inhibit TNF-α (Yan et al., 2007). Homologues of these proteins have been also found in several *L. casei* strains (Yan et al., 2007; Bauerl et al., 2010). The study on the extracellular proteome of *B. animalis* subsp. *lactis* BB-12 revealed six proteins with potential immunogenic effect (e.g., ClpB and Rpf; Gilad et al., 2011). In *L. plantarum* strains, six genetic loci were determined with potential impact on the production of the cytokines IL-10 and IL-12 by PBMCs. These loci included genes which might induce anti- or pro-inflammatory immune responses in the intestine (van Hemert et al., 2010). Genetic loci that might regulate the immune response of DCs were also identified in *L. plantarum* WCFS1 (Meijerink et al., 2010). SlpA has been shown to induce IL-10 production in DCs (Konstantinov et al., 2008) and to intensify immune protection by conferring resistance to infection by *Salmonella enterica* serovar Typhimurium FP1 (Beganovic et al., 2011). Moreover, proteomic analysis of the surface proteins of *Propionibacterium freudenreichii* ITG P20 strain revealed that several SLPs (e.g., InlA, LspA, SlpE, SlpA, SlpB) are contributing factors in the induction of the IL-10 and IL-6 regulatory cytokines (Le Marechal et al., 2014).

Serine protease inhibitor (serpin)-encoding genes (*ser*) are found in several bifidobacteria and they are involved in the inhibition of elastases, components related to intestinal inflammation (Turroni et al., 2010b). It has also been shown that *B. breve* UCC2003 produces a cell surface-associated EPS, encoded by each half of a bidirectional gene cluster, which evokes a weak adaptive immune response and it provides protection against the gut pathogen *Citrobacter rodentium* (Fanning et al., 2012).

Defensins are inducible antimicrobial peptides of the innate immune system, which play an important role in host defenses (Ganz, 2003). It has been demonstrated that the protein flagellin, produced by the probiotic strain *E. coli* Nissle 1917, induces the expression of human β-defensin 2 (hBD-2) by the intestinal epithelium (Schlee et al., 2007). Mutants devoid of the gene responsible for the production of flagellin, presented decreased ability to induce hBD-2.

Our understanding of the mechanisms involved in the modulation of the host’s immune responses by probiotics is far from complete. Even though several genetic and omics studies have shed some light on the relevant mechanisms there is much ground to be covered. Novel developments in meta-transcriptomics and meta-proteomics are expected to speed up research in this field since they will allow the direct study of the interactions between microorganisms and the cell of the host. Currently, it is very difficult to determine *a priori* whether a strain could modulate immune responses (beneficial or not), based solely on sequence data. This is the main reason making it necessary to assess immunomodulatory properties during screening for probiotics with *in vitro* and/or *in vivo* tests.

### PRODUCTION OF ANTIMICROBIAL COMPOUNDS

Probiotic strains often produce an array of antimicrobial compounds. Several of these, like organic acids (e.g., lactic acid) are primary metabolites. In most of these cases, the molecular players of these metabolic pathways for those molecules have been well studied. Other antimicrobial compounds are secondary metabolites, like bacteriocins. The biosynthetic regulons of many bacteriocins have been described and their mode of action has been elucidated. The beneficial effect of bacteriocin-producing probiotic strains against invasive enteropathogens has been well established *in vivo*. It has been demonstrated that the bacteriocin Abp118, produced by *L. salivarius* UCC118, was active against *Li*s*teria monocytogenes* in mice (Corr et al., 2007). High throughput sequencing of bacterial genomes allows the rapid identification of genetic loci related to bacteriocin production and/or immunity. However, bacteriocins should not strictly be considered as antimicrobials. For example it has been demonstrated that six genes of *L. plantarum* WCFS1 associated with the plantaricin biosynthesis and secretion, regulated the production of pro- and anti-inflammatory cytokines of DCs (Meijerink et al., 2010).

### QUORUM SENSING

QS is a communication mechanism among bacterial cells which allows the orchestrated expression of genes within bacterial populations. Its function relies on signaling molecules known as autoinducers. In Gram negative bacteria QS signaling relies on *N*-acylhomoserine lactones (AHL), while in Gram positive bacteria this procedure is dependent upon small cyclic and linear peptides (Waters and Bassler, 2005).

In *L. plantarum* WCFS1 genome an *agr*-like two component regulatory system, encoded by the *lamBDCA* operon, was identified. This operon contained four genes encoding an autoinducing signaling peptide (AIP) modification protein (*lamB*), an AIP (*lamD*), a membrane-located histidine protein kinase (*lamC*) and a cytoplasmic response regulator (*lamA*). This system encodes for a cyclic thiolactone autoinducing peptide (CVGIW) which regulates the adhesion capability of the strain (Sturme et al., 2005). The same strain was shown to carry a second *agr*-like QS system encoded by the *lamKR* operon, which was similar to the *lamBDCA* operon, suggesting analogous function (Fujii et al., 2008). The probiotic strain *E. coli* Nissle 1917 was shown to produce AI-2 molecules (e.g., furanosyl borate diester), involving the *luxS* (autoinducer) gene expression. These molecules were found to influence the induction of anti-inflammatory cytokines in a mouse model of acute colitis (Jacobi et al., 2012). In *L. acidophilus* NCFM, the transcription of the *luxS* gene was notably increased after co-cultivation with live *Listeria monocytogenes* cells, indicating the important role of signaling molecules to the adhesion and the competitive exclusion of pathogens in the GIT (Buck et al., 2009; Moslehi-Jenabian et al., 2011). *B. animalis* subsp. *lactis* BB-12 produces a QS system related peptide (CHWPR). It has been demonstrated that this peptide enhances the expression of two genes in somatic cells, the gene *c-myc*, the deregulation of which has been associated with several forms of cancer, and IL-6, an anti- and pro-inflammatory cytokine (Mitsuma et al., 2008). The role of AI-2 signaling molecule to the adhesive potential of probiotic lactobacilli has also been addressed. It has been established that the disruption of the *luxS* gene reduced significantly the adherence to IECs (Buck et al., 2009).

QS is a very important aspect that may dramatically affect the efficacy of probiotics *in vivo*. Probiotics are entering an already established microbiome, including established biofilms, in which they need to be incorporated. In such an ecological system inter- and intra-species communication is vital to secure temporary colonization of the host, release of antimicrobial compounds and competitive exclusion of pathogens. Evidently, there is an ongoing communication between the microbiome (including any probiotic strain present) and the cells of the host, perhaps beyond immunomodulation. Detailed knowledge about these phenomena may allow us to optimize the probiotic effect.

### PRODUCTION OF NUTRIENTS AND OTHER BENEFICIAL PROCESSES

Production of nutrients in the GIT by probiotic bacteria is an essential process for both the host and the microbiome. *In silico* genome analysis of two *L. reuteri* strains revealed genes responsible for the production of vitamins, essential amino acids, lactate and SCFA (Saulnier et al., 2011). It was shown that both strains hold complete biosynthetic pathways for folate and vitamin B12 and that one of the strains carried also a pathway for the production of vitamin B1. Analysis of the *P. freudenreichii* CIRM-BIA1^T^ genome revealed genes associated with the production of SCFAs and the precursor of menaquinone (vitamin K2), a bifidogenic compound (Falentin et al., 2010). The *fos* gene cluster of *B. animalis* subsp. *lactis* AD011 is another bifidogenic agent, since it is implicated in the processing of health-promoting fructooligosaccharides (Kim et al., 2009). This cluster was shown to have high similarity to the relevant operon described for *B. breve* UCC2003 (Ryan et al., 2005). Furthermore, probiotic bacteria appear to hold a significant role in the modulation of nutrient absorption and in the regulation of the host’s energy balance. Several bifidobacteria possess genes encoding for ABC carbohydrate transporters. The latter contribute to the high consumption rate of specific carbohydrates resulting in high production of acetate, a metabolite which confers protection from enteropathogenic infection (Fukuda et al., 2012). Furthermore, genetic and proteomic analysis of *L. acidophilus* A4 revealed that the catabolite control protein A (*ccpA*) is probably involved in the reduction of total serum cholesterol by influencing the expression of several membrane associated proteins. These proteins may play a role in the adhesion of the cholesterol to the bacterial cells and consequently, in the process of lowering blood cholesterol (Lee et al., 2010).

### FUTURE PERSPECTIVES FOR THE USE OF OMICS IN PROBIOTIC RESEARCH

Several aspects of the molecular mechanisms that underpin probiotic properties have been elucidated. Original studies relied on molecular analysis of single genes and proteins. Over the last decade the advent of omics technologies have allowed the study of probiotic organisms at the whole genome level (Gueimonde and Collado, 2012). Today, metagenomics methodologies are revealing the composition of complex ecosystems and their biology (Qin et al., 2010; Upadrasta et al., 2011). All niches of the human body carry microbiomes that diverge according to the specific compartment, the age and the dietary habits of the individual and many other factors (Ravel et al., 2014). Meta-omics offer for the first time the proper tools for understanding the *in vivo* behavior of probiotics in contrast to simulated conditions involving pure or only a handful of microbial cultures. As omics technologies become cheaper the genome sequencing of microorganisms will become a routine practice. Incorporation of novel functional data through high throughput transcriptomics and proteomics into databases will ultimately facilitate the *in silico* assessment of the probiotic potential of candidate microorganisms. It is too early to speculate whether *in silico* analysis will totally abolish experimental approaches. It is certain though that as our understanding of the molecular biology of probiotic properties improves, we will be able to design more efficient and more sophisticated *in vitro* and *in vivo* tests. Finally the cataloging of the human microbiome has already opened up the door for considering new categories of microorganisms as potential probiotics beyond the usual suspects (i.e., LAB and bifidobacteria).

## CONCLUSION

In this review we presented a detailed overview of the different methodologies employed for the discovery of new probiotic strains. The diversity of screening assays is considerable and their efficacy variable. Some assays are more applicable for screening high numbers of strains while others are more appropriate for validating the probiotic properties of a handful of strains. There is no bulletproof procedure or workflow for selecting probiotics than perhaps the actual testing of candidate strains on the target population (Rijkers et al., 2010). However, considering the limitations of human trials, traditional *in vitro* and *in vivo* assays along with novel omics approaches will remain important. To take full advantage of probiotics for the health of humans a methodological evolution is needed. For example, new “humanized” animal models may be necessary to study host–microbe interactions. Such developments need to go hand in hand with improvements in legislation, ethics, if we want to meet the scientific and technological challenges of probiotic research.

Today, in most parts of the Western world, the acceptance of health claim dossiers is very difficult. In Europe the use of the term “probiotic” has been banned in communications toward the consumer. In the food area, except for a health claim on lactose tolerance for yogurt, none of the 300 bundled health claim dossiers have been approved. It can be hoped that a more profound study of the mechanisms of action and a better understanding of the microbiome functioning and its dynamics with the host, will provide the health claim evaluation panels with the necessary evidence to consider a wider legal acceptance of health claims. While some of the technological evolutions mentioned in this review have the potential to contribute significantly to this, the willingness to consider new types of probiotics, such as *F. prausnitzii* or *A. muciniphila*, currently not yet available on the market, will be crucial.

Not treated in this review is the importance of the production process. With these new, highly anaerobic organisms such as *F. prausnitzii* or *A. muciniphila* in mind, it will be important for probiotic producers to come up with new production processes and modified preservation and administration strategies to guarantee the delivery of active strains to the consumer or patient. As several papers have shown (van Baarlen et al., 2009; Lebeer et al., 2011, 2012; Bron et al., 2012; van Bokhorst-van de Veen et al., 2012) the (industrial) processing of a probiotic preparation has a fundamental impact on the functionality in the host. Viability, the presence or absence of pili, the cell wall condition, the matrix or the growth stage of the probiotic, they all seem to have an important influence on its performance and its interaction with the host. Defining the mechanism of action of a probiotic might therefore also include some critical parameters of the production process.

Without any doubt, the continued development of omics technologies will assist in alleviating the shortages currently faced with the traditional *in vitro* and *in vivo* models. Although it may take a while before we can predict probiotic functionality directly from genomic and metagenomic information, the use of omics approaches to follow up on interesting *in vitro* or *in vivo* observations is very likely going to speed up research progress in the field of probiotics in the near future.

## Conflict of Interest Statement

The authors declare that the research was conducted in the absence of any commercial or financial relationships that could be construed as a potential conflict of interest.

## Footnotes

1 http://ec.europa.eu/research/health/pdf/summary-report-25082010_en.pdf

2 http://www.wageningenur.nl/en/project/Models-of-dietary-effects-on-immune-responses-in-pigs-6.htm

## References

[B1] Abratt V. R. Reid S. J. (2010). “Oxalate-degrading bacteria of the human gut as probiotics in the management of kidney stone disease,” in *Advances in Applied Microbiology*, eds Allen S. S. Laskin I. Geoffrey M. G. (San Diego, CA: Academic Press), 63–87. 10.1016/S0065-2164(10)72003-7 20602988

[B2] Alak J. I. Wolf B. W. Mdurvwa E. G. Pimentel-Smith G. E. Adeyemo O. (1997). Effect of *Lactobacillus reuteri* on intestinal resistance to *Cryptosporidium parvum* infection in a murine model of acquired immunodeficiency syndrome. *J. Infect. Dis.* 175 218–221 10.1093/infdis/175.1.218 8985225

[B3] Al Kassaa I. Hober D. Hamze M. Chihib N. E. Drider D. (2014). Antiviral potential of lactic acid bacteria and their bacteriocins. *Probiotics Antimicrob. Proteins* 6 177–185 10.1007/s12602-014-9162-6 24880436

[B4] Amaretti A. Di Nunzio M. Pompei A. Raimondi S. Rossi M. Bordoni A. (2013). Antioxidant properties of potentially probiotic bacteria: *in vitro* and *in vivo* activities. *Appl. Microbiol. Biotechnol.* 97 809–817 10.1007/s00253-012-4241-7 22790540

[B5] An H. Douillard F. P. Wang G. Zhai Z. Yang J. Song S. (2014). Integrated transcriptomic and proteomic analysis of the bile stress response in a centenarian-originated probiotic *Bifidobacterium longum* BBMN68. *Mol. Cell Proteomics* 13 2558–2572 10.1074/mcp.M114.039156 24965555 PMC4188986

[B6] Archambaud C. Nahori M. A. Soubigou G. Becavin C. Laval L. Lechat P. (2012). Impact of lactobacilli on orally acquired listeriosis. *Proc. Natl. Acad. Sci. U.S.A.* 109 16684–16689 10.1073/pnas.1212809109 23012479 PMC3478606

[B7] Argyri A. A. Zoumpopoulou G. Karatzas K. A. Tsakalidou E. Nychas G. J. Panagou E. Z. (2013). Selection of potential probiotic lactic acid bacteria from fermented olives by *in vitro* tests. *Food Microbiol.* 33 282–291 10.1016/j.fm.2012.10.005 23200662

[B8] Ashida N. Yanagihara S. Shinoda T. Yamamoto N. (2011). Characterization of adhesive molecule with affinity to Caco-2 cells in *Lactobacillus acidophilus* by proteome analysis. *J. Biosci. Bioeng.* 112 333–337 10.1016/j.jbiosc.2011.06.001 21763196

[B9] Aso Y. Akaza H. Kotake T. Tsukamoto T. Imai K. Naito S. (1995). Preventive effect of a *Lactobacillus casei* preparation on the recurrence of superficial bladder cancer in a double-blind trial. The BLP Study Group. *Eur. Urol.* 27 104–109. 7744150 10.1159/000475138

[B10] Azcarate-Peril M. A. Altermann E. Goh Y. J. Tallon R. Sanozky-Dawes R. B. Pfeiler E. A. (2008). Analysis of the genome sequence of *Lactobacillus gasseri* ATCC 33323 reveals the molecular basis of an autochthonous intestinal organism. *Appl. Environ. Microbiol.* 74 4610–4625 10.1128/AEM.00054-08 18539810 PMC2519322

[B11] Baker D. G. (1998). Natural pathogens of laboratory mice, rats, and rabbits and their effects on research. *Clin. Microbiol. Rev.* 11 231–266. 9564563 10.1128/cmr.11.2.231 PMC106832

[B12] Bao Y. Zhang Y. Zhang Y. Liu Y. Wang S. Dong X. (2010). Screening of potential probiotic properties of *Lactobacillus fermentum* isolated from traditional dairy products. *Food Control* 21 695–701 10.1016/j.foodcont.2009.10.010

[B13] Barada K. A. Mourad F. H. Sawah S. I. Khoury C. Safieh-Garabedian B. Nassar C. F. (2007). Up-regulation of nerve growth factor and interleukin-10 in inflamed and non-inflamed intestinal segments in rats with experimental colitis. *Cytokine* 37 236–245 10.1016/j.cyto.2007.04.005 17517520

[B14] Barc M. C. Charrin-Sarnel C. Rochet V. Bourlioux F. Sandre C. Boureau H. (2008). Molecular analysis of the digestive microbiota in a gnotobiotic mouse model during antibiotic treatment: influence of *Saccharomyces boulardii*. *Anaerobe* 14 229–233 10.1016/j.anaerobe.2008.04.003 18511310

[B15] Bauerl C. Perez-Martinez G. Yan F. Polk D. B. Monedero V. (2010). Functional analysis of the p40 and p75 proteins from *Lactobacillus casei* BL23. *J. Mol. Microbiol. Biotechnol.* 19 231–241 10.1159/000322233 21178363 PMC3019367

[B16] Beganovic J. Frece J. Kos B. Lebos Pavunc A. Habjanic K. Suskovic J. (2011). Functionality of the S-layer protein from the probiotic strain *Lactobacillus helveticus* M92. *Antonie Van Leeuwenhoek* 100 43–53 10.1007/s10482-011-9563-4 21327475

[B17] Begley M. Gahan C. G. Hill C. (2005). The interaction between bacteria and bile. *FEMS Microbiol. Rev.* 29 625–651 10.1016/j.femsre.2004.09.003 16102595

[B18] Bernardeau M. Guguen M. Vernoux J. P. (2006). Beneficial lactobacilli in food and feed: long-term use, biodiversity and proposals for specific and realistic safety assessments. *FEMS Microbiol. Rev.* 30 487–513 10.1111/j.1574-6976.2006.00020.x 16774584

[B19] Boekhorst J. Helmer Q. Kleerebezem M. Siezen R. J. (2006). Comparative analysis of proteins with a mucus-binding domain found exclusively in lactic acid bacteria. *Microbiology* 152 273–280 10.1099/mic.0.28415-0 16385136

[B20] Boffa L. C. Lupton J. R. Mariani M. R. Ceppi M. Newmark H. L. Scalmati A. (1992). Modulation of colonic epithelial cell proliferation, histone acetylation, and luminal short chain fatty acids by variation of dietary fiber (wheat bran) in rats. *Cancer Res.* 52 5906–5912. 1327519

[B21] Borchers A. T. Selmi C. Meyers F. J. Keen C. L. Gershwin M. E. (2009). Probiotics and immunity. *J. Gastroenterol.* 44 26–46 10.1007/s00535-008-2296-0 19159071

[B22] Borenshtein D. Mcbee M. E. Schauer D. B. (2008). Utility of the *Citrobacter rodentium* infection model in laboratory mice. *Curr. Opin. Gastroenterol.* 24 32–37 10.1097/MOG.0b013e3282f2b0fb 18043230

[B23] Botta C. Langerholc T. Cencic A. Cocolin L. (2014). *In vitro* selection and characterization of new probiotic candidates from table olive microbiota. *PLoS ONE* 9:e94457 10.1371/journal.pone.0094457 PMC3979845 24714329

[B24] Bove P. Gallone A. Russo P. Capozzi V. Albenzio M. Spano G. (2012). Probiotic features of *Lactobacillus plantarum* mutant strains. *Appl. Microbiol. Biotechnol.* 96 431–441 10.1007/s00253-012-4031-2 22573266

[B25] Bover-Cid S. Holzapfel W. H. (1999). Improved screening procedure for biogenic amine production by lactic acid bacteria. *Int. J. Food. Microbiol.* 53 33–41 10.1016/S0168-1605(99)00152-X 10598112

[B26] Bron P. Van Bokhorst-Van De Veen H. Wels M. Kleerebezem M. (2011). “Engineering robust lactic acid bacteria,” in *Stress Responses of Lactic Acid Bacteria*, eds Tsakalidou E. Papadimitriou K. (New York: Springer), 369–394 10.1007/978-0-387-92771-8_16

[B27] Bron P. A. Wels M. Bongers R. S. Van Bokhorst-Van De Veen H. Wiersma A. Overmars L. (2012). Transcriptomes reveal genetic signatures underlying physiological variations imposed by different fermentation conditions in *Lactobacillus plantarum*. *PLoS ONE* 7:e38720 10.1371/journal.pone.0038720 PMC3389018 22802930

[B28] Buck B. L. Azcarate-Peril M. A. Klaenhammer T. R. (2009). Role of autoinducer-2 on the adhesion ability of *Lactobacillus acidophilus*. *J. Appl. Microbiol.* 107 269–279 10.1111/j.1365-2672.2009.04204.x 19302300

[B29] Burns A. J. Rowland I. R. (2004). Antigenotoxicity of probiotics and prebiotics on faecal water-induced DNA damage in human colon adenocarcinoma cells. *Mutat. Res.* 551 233–243 10.1016/j.mrfmmm.2004.03.010 15225596

[B30] Campieri C. Campieri M. Bertuzzi V. Swennen E. Matteuzzi D. Stefoni S. (2001). Reduction of oxaluria after an oral course of lactic acid bacteria at high concentration. *Kidney Int.* 60 1097–1105 10.1046/j.1523-1755.2001.0600031097.x 11532105

[B31] Candela M. Biagi E. Centanni M. Turroni S. Vici M. Musiani F. (2009). Bifidobacterial enolase, a cell surface receptor for human plasminogen involved in the interaction with the host. *Microbiology* 155 3294–3303 10.1099/mic.0.028795-0 19574304

[B32] Cani P. D. Bibiloni R. Knauf C. Waget A. Neyrinck A. M. Delzenne N. M. (2008). Changes in gut microbiota control metabolic endotoxemia-induced inflammation in high-fat diet-induced obesity and diabetes in mice. *Diabetes Metab. Res. Rev.* 57 1470–1481 10.2337/db07-1403 18305141

[B33] Cani P. Everard A. Belzer C. De V. W. (2014). *Use of Akkermansia for Treating Metabolic Disorders*. Patent no. WO2014075745A1.

[B34] Cani P. D. Lecourt E. Dewulf E. M. Sohet F. M. Pachikian B. D. Naslain D. (2009). Gut microbiota fermentation of prebiotics increases satietogenic and incretin gut peptide production with consequences for appetite sensation and glucose response after a meal. *Am. J. Clin. Nutr.* 90 1236–1243 10.3945/ajcn.2009.28095 19776140

[B35] Cani P. D. Van Hul M. (2015). Novel opportunities for next-generation probiotics targeting metabolic syndrome. *Curr. Opin. Biotechnol.* 32 21–27 10.1016/j.copbio.2014.10.006 25448228

[B36] Castro M. S. Molina M. A. Di Sciullo P. Azpiroz M. B. Leocata Nieto F. Sterin-Speziale N. B. (2010). Beneficial activity of *Enterococcus faecalis* CECT7121 in the anti-lymphoma protective response. *J. Appl. Microbiol.* 109 1234–1243 10.1111/j.1365-2672.2010.04747.x 20477887

[B37] Cencic A. Langerholc T. (2010). Functional cell models of the gut and their applications in food microbiology-a review. *Int. J. Food Microbiol.* 141(Suppl. 1), S4–S14 10.1016/j.ijfoodmicro.2010.03.026 20444515 PMC7173225

[B38] Céspedes M. Cárdenas P. Staffolani M. Ciappini M. C. Vinderola G. (2013). Performance in nondairy drinks of probiotic *L. casei* strains usually employed in dairy products. *J. Food Sci.* 78 M756–M762 10.1111/1750-3841.12092 23527588

[B39] Challa A. Rao D. R. Chawan C. B. Shackelford L. (1997). *Bifidobacterium longum* and lactulose suppress azoxymethane-induced colonic aberrant crypt foci in rats. *Carcinogenesis* 18 517–521 10.1093/carcin/18.3.517 9067551

[B40] Charteris W. P. Kelly P. M. Morelli L. Collins J. K. (1998). Development and application of an *in vitro* methodology to determine the transit tolerance of potentially probiotic *Lactobacillus* and *Bifidobacterium* species in the upper human gastrointestinal tract. *J. Appl. Microbiol.* 84 759–768 10.1046/j.1365-2672.1998.00407.x 9674129

[B41] Chen C. C. Louie S. Shi H. N. Walker W. A. (2005). Preinoculation with the probiotic *Lactobacillus acidophilus* early in life effectively inhibits murine *Citrobacter rodentium* colitis. *Pediatr. Res.* 58 1185–1191 10.1203/01.pdr.0000183660.39116.83 16306191

[B42] Chen X. Fruehauf J. Goldsmith J. D. Xu H. Katchar K. K. Koon H. W. (2009). *Saccharomyces boulardii* inhibits EGF receptor signaling and intestinal tumor growth in Apc^min^ mice. *Gastroenterology* 137 914–923 10.1053/j.gastro.2009.05.050 19482027 PMC2777664

[B43] Chen X. Katchar K. Goldsmith J. D. Nanthakumar N. Cheknis A. Gerding D. N. (2008). A mouse model of *Clostridium difficile*-associated disease. *Gastroenterology* 135 1984–1992 10.1053/j.gastro.2008.09.002 18848941

[B44] Chenoll E. Casinos B. Bataller E. Astals P. Echevarría J. Iglesias J. R. (2011). Novel probiotic *Bifidobacterium bifidum* CECT 7366 strain active against the pathogenic bacterium *Helicobacter pylori*. *Appl. Environ. Microbiol.* 77 1335–1343 10.1128/AEM.01820-10 21169430 PMC3067243

[B45] Choi S. S. Kim Y. Han K. S. You S. Oh S. Kim S. H. (2006). Effects of *Lactobacillus* strains on cancer cell proliferation and oxidative stress *in vitro*. *Lett. Appl. Microbiol.* 42 452–458 10.1111/j.1472-765X.2006.01913.x 16620202

[B46] Chong E. (2014). A potential role of probiotics in colorectal cancer prevention: review of possible mechanisms of action. *World J. Microbiol. Biotechnol.* 30 351–374 10.1007/s11274-013-1499-6 24068536

[B47] Claes I. J. De Keersmaecker S. C. Vanderleyden J. Lebeer S. (2011). Lessons from probiotic-host interaction studies in murine models of experimental colitis. *Mol. Nutr. Food Res.* 55 1441–1453 10.1002/mnfr.201100139 21796777

[B48] Collado M. Meriluoto J. Salminen S. (2008). Adhesion and aggregation properties of probiotic and pathogen strains. *Eur. Food Res. Technol.* 226 1065–1073 10.1007/s00217-007-0632-x

[B49] Collado M. C. Meriluoto J. Salminen S. (2007). Measurement of aggregation properties between probiotics and pathogens: *in vitro* evaluation of different methods. *J. Microbiol. Methods* 71 71–74 10.1016/j.mimet.2007.07.005 17719109

[B50] Collins J. W. Chervaux C. Raymond B. Derrien M. Brazeilles R. Kosta A. (2014). Fermented dairy products modulate *Citrobacter rodentium*-induced colonic hyperplasia. *J. Infect. Dis.* 210 1029–1041 10.1093/infdis/jiu205 24706936 PMC4157696

[B51] Coman M. M. Verdenelli M. C. Cecchini C. Silvi S. Orpianesi C. Boyko N. (2014). *In vitro* evaluation of antimicrobial activity of *Lactobacillus rhamnosus* IMC 501®, *Lactobacillus paracasei* IMC 502® and SYNBIO® against pathogens. *J. Appl. Microbiol.* 117 518–527 10.1111/jam.12544 24836638

[B52] Commane D. M. Shortt C. T. Silvi S. Cresci A. Hughes R. M. Rowland I. R. (2005). Effects of fermentation products of pro- and prebiotics on trans-epithelial electrical resistance in an *in vitro* model of the colon. *Nutr. Cancer* 51 102–109 10.1207/s15327914nc5101-14 15749636

[B53] Comuzzie A. G. Allison D. B. (1998). The search for human obesity genes. *Science* 280 1374–1377 10.1126/science.280.5368.1374 9603720

[B54] Conway P. L. Gorbach S. L. Goldin B. R. (1987). Survival of lactic acid bacteria in the human stomach and adhesion to intestinal cells. *J. Dairy Sci.* 70 1–12 10.3168/jds.S0022-0302(87)79974-3 3106442

[B55] Cook M. T. Tzortzis G. Charalampopoulos D. Khutoryanskiy V. V. (2012). Microencapsulation of probiotics for gastrointestinal delivery. *J. Control Release* 162 56–67 10.1016/j.jconrel.2012.06.003 22698940

[B56] Corr S. C. Li Y. Riedel C. U. O’toole P. W. Hill C. Gahan C. G. (2007). Bacteriocin production as a mechanism for the antiinfective activity of *Lactobacillus salivarius* UCC118. *Proc. Natl. Acad. Sci. U.S.A.* 104 7617–7621 10.1073/pnas.0700440104 17456596 PMC1863472

[B57] Corthesy B. Gaskins H. R. Mercenier A. (2007). Cross-talk between probiotic bacteria and the host immune system. *J. Nutr.* 137 781S–790S. 17311975 10.1093/jn/137.3.781S

[B58] Cousin F. J. Jouan-Lanhouet S. Dimanche-Boitrel M.-T. Corcos L. Jan G. (2012). Milk fermented by *Propionibacterium freudenreichii* induces apoptosis of HGT-1 human gastric cancer cells. *PLoS ONE* 7:e31892 10.1371/journal.pone.0031892 PMC3307715 22442660

[B59] Cresci G. Nagy L. E. Ganapathy V. (2013). *Lactobacillus* GG and tributyrin supplementation reduce antibiotic-induced intestinal injury. *J. Parenter. Enteral Nutr.* 37 763–774 10.1177/0148607113486809 PMC3818407 23630018

[B60] Cross M. L. (2002). Microbes versus microbes: immune signals generated by probiotic lactobacilli and their role in protection against microbial pathogens. *FEMS Immunol. Med. Microbiol.* 34 245–253 10.1111/j.1574-695X.2002.tb00632.x 12443824

[B61] Dashkevicz M. P. Feighner S. D. (1989). Development of a differential medium for bile salt hydrolase-active *Lactobacillus* spp. *Appl. Environ. Microbiol.* 55 11–16. 2705765 10.1128/aem.55.1.11-16.1989 PMC184046

[B62] De Bruyn F. Beauprez J. Maertens J. Soetaert W. De Mey M. (2013). Unraveling the Leloir pathway of *Bifidobacterium bifidum*: significance of the uridylyltransferases. *Appl. Environ. Microbiol.* 79 7028–7035 10.1128/AEM.02460-13 24014529 PMC3811521

[B63] de LeBlanc Ade M. Castillo N. A. Perdigon G. (2010). Anti-infective mechanisms induced by a probiotic *Lactobacillus* strain against *Salmonella enterica* serovar Typhimurium infection. *Int. J. Food Microbiol.* 138 223–231 10.1016/j.ijfoodmicro.2010.01.020 20193971

[B64] Delgado S. O’sullivan E. Fitzgerald G. Mayo B. (2007). Subtractive screening for probiotic properties of *Lactobacillus* species from the human gastrointestinal tract in the search for new probiotics. *J. Food Sci.* 72 M310–M315 10.1111/j.1750-3841.2007.00479.x 17995611

[B65] Del Re B. Sgorbati B. Miglioli M. Palenzona D. (2000). Adhesion, autoaggregation and hydrophobicity of 13 strains of *Bifidobacterium longum*. *Lett. Appl. Microbiol.* 31 438–442 10.1046/j.1365-2672.2000.00845.x 11123552

[B66] Derrien M. Van Passel M. W. Van De Bovenkamp J. H. Schipper R. G. De Vos W. M. Dekker J. (2010). Mucin-bacterial interactions in the human oral cavity and digestive tract. *Gut Microbes* 1 254–268 10.4161/gmic.1.4.12778 21327032 PMC3023607

[B67] Douillard F. P. Ribbera A. Jarvinen H. M. Kant R. Pietila T. E. Randazzo C. (2013). Comparative genomic and functional analysis of *Lactobacillus casei* and *Lactobacillus rhamnosus* strains marketed as probiotics. *Appl. Environ. Microbiol.* 79 1923–1933 10.1128/AEM.0346712 23315726 PMC3592221

[B68] Duangjitcharoen Y. Kantachote D. Prasitpuripreecha C. Peerajan S. Chaiyasut C. (2014). Selection and characterisation of probiotic lactic acid bacteria with heterocyclic amine binding and nitrosamine degradation properties. *J. Appl. Pharm. Sci.* 4 014–023 10.7324/JAPS.2014.40703

[B69] Duncan S. H. Lobley G. E. Holtrop G. Ince J. Johnstone A. M. Louis P. (2008). Human colonic microbiota associated with diet, obesity and weight loss. *Int. J. Obes. (Lond.)* 32 1720–1724 10.1038/ijo.2008.155 18779823

[B70] Eaton K. A. Honkala A. Auchtung T. A. Britton R. A. (2011). Probiotic *Lactobacillus reuteri* ameliorates disease due to enterohemorrhagic *Escherichia coli* in germfree mice. *Infect. Immun.* 79 185–191 10.1128/IAI.00880-10 20974822 PMC3019869

[B71] Ebel B. Lemetais G. Beney L. Cachon R. Sokol H. Langella P. (2014). Impact of probiotics on risk factors for cardiovascular diseases. *Crit. Rev. Food Sci. Nutr.* 54 175–189 10.1080/10408398.2011.579361 24188267

[B72] Eck P. Friel J. (2013). Should probiotics be considered as vitamin supplements? *Vitam*. *Miner.* 3 e124. 10.4172/vms.1000e124

[B73] EFSA. (2008). 2008 Annual report on pesticide residues according to Article 32 of Regulation (EC) No 396/2005. *EFSA J. 2010.* 8:1646.

[B74] Eutamene H. Lamine F. Chabo C. Theodorou V. Rochat F. Bergonzelli G. E. (2007). Synergy between *Lactobacillus paracasei* and its bacterial products to counteract stress-induced gut permeability and sensitivity increase in rats. *J. Nutr.* 137 1901–1907. 17634262 10.1093/jn/137.8.1901

[B75] Evans N. P. Misyak S. A. Schmelz E. M. Guri A. J. Hontecillas R. Bassaganya-Riera J. (2010). Conjugated linoleic acid ameliorates inflammation-induced colorectal cancer in mice through activation of PPARgamma. *J. Nutr.* 140 515–521 10.3945/jn.109.115642 20089779 PMC2821885

[B76] Everard A. Belzer C. Geurts L. Ouwerkerk J. P. Druart C. Bindels L. B. (2013). Cross-talk between *Akkermansia muciniphila* and intestinal epithelium controls diet-induced obesity. *Proc. Natl. Acad. Sci. U.S.A.* 110 9066–9071 10.1073/pnas.1219451110 23671105 PMC3670398

[B77] Ewaschuk J. B. Diaz H. Meddings L. Diederichs B. Dmytrash A. Backer J. (2008). Secreted bioactive factors from *Bifidobacterium infantis* enhance epithelial cell barrier function. *Am. J. Physiol. Gastrointest. Liver Physiol.* 295 G1025–G1034 10.1152/ajpgi.90227.2008 18787064

[B78] Ewaschuk J. B. Walker J. W. Diaz H. Madsen K. L. (2006). Bioproduction of conjugated linoleic acid by probiotic bacteria occurs *in vitro* and *in vivo* in mice. *J. Nutr.* 136 1483–1487. 16702308 10.1093/jn/136.6.1483

[B79] Falentin H. Deutsch S. M. Jan G. Loux V. Thierry A. Parayre S. (2010). The complete genome of *Propionibacterium freudenreichii* CIRM-BIA1T, a hardy actinobacterium with food and probiotic applications. *PLoS ONE* 5:e11748 10.1371/journal.pone.0011748 PMC2909200 20668525

[B80] Fanning S. Hall L. J. Cronin M. Zomer A. Macsharry J. Goulding D. (2012). Bifidobacterial surface-exopolysaccharide facilitates commensal-host interaction through immune modulation and pathogen protection. *Proc. Natl. Acad. Sci. U.S.A.* 109 2108–2113 10.1073/pnas.1115621109 22308390 PMC3277520

[B81] Faridnia F. Hussin A. S. Saari N. Mustafa S. Yee L. Y. Manap M. Y. (2010). *In vitro* binding of mutagenic heterocyclic aromatic amines by *Bifidobacterium pseudocatenulatum* G4. *Benef. Microbes* 1 149–154 10.3920/BM2009.0035 21831754

[B82] Fasseas M. K. Fasseas C. Mountzouris K. C. Syntichaki P. (2013). Effects of *Lactobacillus salivarius*, *Lactobacillus reuteri*, and *Pediococcus acidilactici* on the nematode *Caenorhabditis elegans* include possible antitumor activity. *Appl. Microbiol. Biotechnol.* 97 2109–2118 10.1007/s00253-012-4357-9 22923095

[B83] Ferreira A. B. De Oliveira M. N. Freitas F. S. Alfenas-Zerbini P. Da Silva D. F. De Queiroz M. V. (2013). Increased expression of *c/p* genes in *Lactobacillus delbrueckii* UFV H2b20 exposed to acid stress and bile salts. *Benef. Microbes* 4 367–374 10.3920/BM2013.0022 24311319

[B84] Ferreira C. L. Grześkowiak L. Collado M. C. Salminen S. (2011). *In vitro* evaluation of *Lactobacillus gasseri* strains of infant origin on adhesion and aggregation of specific pathogens. *J. Food Prot.* 74 1482–1487 10.4315/0362-028X.JFP-11-074 21902917

[B85] Fitzpatrick L. R. Small J. S. Greene W. H. Karpa K. D. Keller D. (2011). *Bacillus coagulans* GBI-30 (BC30) improves indices of *Clostridium difficile*-induced colitis in mice. *Gut Pathog.* 3 16 10.1186/1757-4749-3-16 PMC3212889 22014083

[B86] Fleming A. Jankowski J. Goldsmith P. (2010). *In vivo* analysis of gut function and disease changes in a zebrafish larvae model of inflammatory bowel disease: a feasibility study. *Inflamm. Bowel Dis.* 16 1162–1172 10.1002/ibd.21200 20128011

[B87] Foligne B. Deutsch S. M. Breton J. Cousin F. J. Dewulf J. Samson M. (2010a). Promising immunomodulatory effects of selected strains of dairy propionibacteria as evidenced *in vitro* and in *vivo*. *Appl. Environ. Microbiol.* 76 8259–8264 10.1128/AEM.01976-10 20971874 PMC3008228

[B88] Foligne B. Dewulf J. Vandekerckove P. Pignede G. Pot B. (2010b). Probiotic yeasts: anti-inflammatory potential of various non-pathogenic strains in experimental colitis in mice. *World J. Gastroenterol.* 16 2134–2145 10.3748/wjg.v16.i17.2134 20440854 PMC2864839

[B89] Foligne B. Nutten S. Grangette C. Dennin V. Goudercourt D. Poiret S. (2007). Correlation between *in vitro* and *in vivo* immunomodulatory properties of lactic acid bacteria. *World J. Gastroenterol.* 13 236–243 10.3748/wjg.v13.i2.236 17226902 PMC4065951

[B90] Fontana L. Bermudez-Brito M. Plaza-Diaz J. Munoz-Quezada S. Gil A. (2013). Sources, isolation, characterisation, and evaluation of probiotics. *Br. J. Nutr.* 109(Suppl. 2), S35–S50 10.1017/S0007114512004011 23360880

[B91] Foroni E. Serafini F. Amidani D. Turroni F. He F. Bottacini F. (2011). Genetic analysis and morphological identification of pilus-like structures in members of the genus *Bifidobacterium*. *Microb. Cell Fact.* 10(Suppl. 1), S16 10.1186/1475-2859-10-S1-S16 PMC3231923 21995649

[B92] Fujii T. Ingham C. Nakayama J. Beerthuyzen M. Kunuki R. Molenaar D. (2008). Two homologous Agr-like quorum-sensing systems cooperatively control adherence, cell morphology, and cell viability properties in *Lactobacillus plantarum* WCFS1. *J. Bacteriol.* 190 7655–7665 10.1128/JB.01489 18805979 PMC2583610

[B93] Fujiwara D. Inoue S. Wakabayashi H. Fujii T. (2004). The anti-allergic effects of lactic acid bacteria are strain dependent and mediated by effects on both Th1/Th2 cytokine expression and balance. *Int. Arch. Allergy Immunol.* 135 205–215 10.1159/000081305 15467373

[B94] Fukuda S. Toh H. Taylor T. D. Ohno H. Hattori M. (2012). Acetate-producing bifidobacteria protect the host from enteropathogenic infection via carbohydrate transporters. *Gut Microbes* 3 449–454 10.4161/gmic.21214 22825494

[B95] Furrie E. (2005). Probiotics and allergy. *Proc. Nutr. Soc.* 64 465–469 10.1079/PNS2005466 16313688

[B96] Gagnon M. Zihler A. Chassard C. Lacroix C. (2011). “Ecology of probiotics and enteric protection,” in *Probiotic Bacteria and Enteric Infections*, eds Malago J. J. Koninkx J. F. J. G. Marinsek-Logar R. (Netherlands: Springer), 65–85 10.1007/978-94-007-0386-5_3

[B97] Ganz T. (2003). Defensins: antimicrobial peptides of innate immunity. *Nat. Rev. Immunol.* 3 710–720 10.1038/nri1180 12949495

[B98] García-Cayuela T. Korany A. M. Bustos I. Gómez De Cadiñanos L. P. Requena T. Peláez C. (2014). Adhesion abilities of dairy *Lactobacillus plantarum* strains showing an aggregation phenotype. *Food Res. Int.* 57 44–50 10.1016/j.foodres.2014.01.010

[B99] Garrido D. Kim J. H. German J. B. Raybould H. E. Mills D. A. (2011). Oligosaccharide binding proteins from *Bifidobacterium longum* subsp. *infantis* reveal a preference for host glycans. *PLoS ONE* 6:e17315 10.1371/journal.pone.0017315 PMC3057974 21423604

[B100] Gepts W. Lecompte P. M. (1981). The pancreatic islets in diabetes. *Am. J. Med.* 70 105–115 10.1016/0002-9343(81)90417-4 7006384

[B101] Gilad O. Svensson B. Viborg A. H. Stuer-Lauridsen B. Jacobsen S. (2011). The extracellular proteome of *Bifidobacterium animalis* subsp. *lactis* BB-12 reveals proteins with putative roles in probiotic effects. *Proteomics* 11 2503–2514 10.1002/pmic.201000716 21598393

[B102] Gilbert J. A. Krajmalnik-Brown R. Porazinska D. L. Weiss S. J. Knight R. (2013). Toward effective probiotics for autism and other neurodevelopmental disorders. *Cell* 155 1446–1448 10.1016/j.cell.2013.11.035 24360269 PMC4166551

[B103] Gill H. Grover S. Batish V. Gill P. (2009). “Immunological effects of probiotics and their significance to human health,” in *Prebiotics and Probiotics Science and Technology*, eds Charalampopoulos D. Rastall R. (New York: Springer), 901–948 10.1007/978-0-387-79058-9_23

[B104] Gioacchini G. Giorgini E. Olivotto I. Maradonna F. Merrifield D. L. Carnevali O. (2014). The influence of probiotics on zebrafish *Danio rerio* innate immunity and hepatic stress. *Zebrafish* 11 98–106 10.1089/zeb.2013.0932 24564619

[B105] Goldin B. R. Gorbach S. L. (1984). Alterations of the intestinal microflora by diet, oral antibiotics, and *Lactobacillus*: decreased production of free amines from aromatic nitro compounds, azo dyes, and glucuronides. *J. Natl. Cancer Inst.* 73 689–695. 6433097

[B106] Gorbach S. L. Goldin B. R. (1992). Nutrition and the gastrointestinal microflora. *Nutr. Rev.* 50 378–381 10.1111/j.1753-4887.1992.tb02485.x 1488172

[B107] Grompone G. Martorell P. Llopis S. Gonzalez N. Genoves S. Mulet A. P. (2012). Anti-inflammatory *Lactobacillus rhamnosus* CNCM I-3690 strain protects against oxidative stress and increases lifespan in *Caenorhabditis elegans*. *PLoS ONE* 7:e52493 10.1371/journal.pone.0052493 PMC3530454 23300685

[B108] Gueimonde M. Collado M. C. (2012). Metagenomics and probiotics. *Clin. Microbiol. Infect.* 18(Suppl. 4), 32–34 10.1111/j.1469-0691.2012.03873.x 22647045

[B109] Gueimonde M. Garrigues C. Van Sinderen D. De Los Reyes-Gavilan C. G. Margolles A. (2009). Bile-inducible eﬄux transporter from *Bifidobacterium longum* NCC2705, conferring bile resistance. *Appl. Environ. Microbiol.* 75 3153–3160 10.1128/AEM.00172-09 19304838 PMC2681658

[B110] Guo X. H. Kim J. M. Nam H. M. Park S. Y. Kim J. M. (2010). Screening lactic acid bacteria from swine origins for multistrain probiotics based on *in vitro* functional properties. *Anaerobe* 16 321–326 10.1016/j.anaerobe.2010.03.006 20304081

[B111] Halász A. Baráth Á. Simon-Sarkadi L. Holzapfel W. (1994). Biogenic amines and their production by microorganisms in food. *Trends Food Sci*. *Technol.* 5 42–49 10.1016/0924-2244(94)90070-1

[B112] Halttunen T. Salminen S. Tahvonen R. (2007). Rapid removal of lead and cadmium from water by specific lactic acid bacteria. *Int. J. Food Microbiol.* 114 30–35 10.1016/j.ijfoodmicro.2006.10.040 17184867

[B113] Hamon E. Horvatovich P. Izquierdo E. Bringel F. Marchioni E. Aoude-Werner D. (2011). Comparative proteomic analysis of *Lactobacillus plantarum* for the identification of key proteins in bile tolerance. *BMC Microbiol.* 11:63 10.1186/1471-2180-11-63 PMC3073879 21447177

[B114] Hamon E. Horvatovich P. Marchioni E. Aoude-Werner D. Ennahar S. (2014). Investigation of potential markers of acid resistance in *Lactobacillus plantarum* by comparative proteomics. *J. Appl. Microbiol.* 116 134–144 10.1111/jam.12339 24016102

[B115] Hapfelmeier S. Hardt W. D. (2005). A mouse model for *S. typhimurium*-induced enterocolitis. *Trends Microbiol.* 13 497–503 10.1016/j.tim.2005.08.008 16140013

[B116] Harty D. W. Oakey H. J. Patrikakis M. Hume E. B. Knox K. W. (1994). Pathogenic potential of lactobacilli. *Int. J. Food Microbiol.* 24 179–189 10.1016/0168-1605(94)90117-1 7703012

[B117] Haukioja A. (2010). Probiotics and oral health. *Eur. J. Dent.* 4 348–355. 20613927 PMC2897872

[B118] He Q. Wang L. Wang F. Wang C. Tang C. Li Q. (2013). Microbial fingerprinting detects intestinal microbiota dysbiosis in Zebrafish models with chemically-induced enterocolitis. *BMC Microbiol.* 13:289 10.1186/1471-2180-13-289 PMC4029296 24325678

[B119] Helm R. M. Burks A. W. (2002). Animal models of food allergy. *Curr. Opin. Allergy Clin. Immunol.* 2 541–546 10.1097/01.all.0000044541.45448.bb 14752339

[B120] Henao-Mejia J. Elinav E. Jin C. Hao L. Mehal W. Z. Strowig T. (2012). Inflammasome-mediated dysbiosis regulates progression of NAFLD and obesity. *Nature* 482 179–185 10.1038/nature10809 22297845 PMC3276682

[B121] Hickson M. (2011). Probiotics in the prevention of antibiotic-associated diarrhoea and *Clostridium difficile* infection. *Therap. Adv. Gastroenterol.* 4 185–197 10.1177/1756283X11399115 PMC3105609 21694803

[B122] Hildebrandt M. A. Hoffmann C. Sherrill-Mix S. A. Keilbaugh S. A. Hamady M. Chen Y. Y. (2009). High-fat diet determines the composition of the murine gut microbiome independently of obesity. *Gastroenterology* 137 1716e1-2 – 1724.e1-2. 10.1053/j.gastro.2009.08.042 PMC2770164 19706296

[B123] Hsiao E. Y. Mcbride S. W. Hsien S. Sharon G. Hyde E. R. Mccue T. (2013). Microbiota modulate behavioral and physiological abnormalities associated with neurodevelopmental disorders. *Cell* 155 1451–1463 10.1016/j.cell.2013.11.024 24315484 PMC3897394

[B124] Hughes D. B. Hoover D. G. (1995). Viability and enzymatic activity of bifidobacteria in milk. *J. Dairy Sci.* 78 268–276 10.3168/jds.S0022-0302(95)76634-6 7745146

[B125] Hummel K. P. Dickie M. M. Coleman D. L. (1966). Diabetes, a new mutation in the mouse. *Science* 153 1127–1128 10.1126/science.153.3740.1127 5918576

[B126] Ichinohe T. Pang I. K. Kumamoto Y. Peaper D. R. Ho J. H. Murray T. S. (2011). Microbiota regulates immune defense against respiratory tract influenza a virus infection. *Proc. Natl. Acad. Sci. U.S.A.* 108 5354–5359 10.1073/pnas.1019378108 21402903 PMC3069176

[B127] Ingalls A. M. Dickie M. M. Snell G. D. (1950). Obese, a new mutation in the house mouse. *J. Hered.* 41 317–318 14824537 10.1093/oxfordjournals.jhered.a106073

[B128] Ito M. Kobayashi K. Nakahata T. (2008). “NOD/Shi-scidIL2rγ^null^ (NOG) mice more appropriate for humanized mouse models,” in *Humanized Mice,* eds Nomura T. Watanabe T. Habu S. (Berlin: Springer), 53–76. 10.1007/978-3-540-75647-7_3 18481452

[B129] Izquierdo E. Horvatovich P. Marchioni E. Aoude-Werner D. Sanz Y. Ennahar S. (2009). 2-DE and MS analysis of key proteins in the adhesion of *Lactobacillus plantarum*, a first step toward early selection of probiotics based on bacterial biomarkers. *Electrophoresis* 30 949–956 10.1002/elps.200800399 19309013

[B130] Jacobi C. A. Grundler S. Hsieh C. J. Frick J. S. Adam P. Lamprecht G. (2012). Quorum sensing in the probiotic bacterium *Escherichia coli* Nissle 1917 (Mutaflor) - evidence that furanosyl borate diester (AI-2) is influencing the cytokine expression in the DSS colitis mouse model. *Gut Pathog.* 4:8 10.1186/1757-4749-4-8 PMC3480846 22862922

[B131] Jacobsen C. N. Rosenfeldt Nielsen V. Hayford A. E. Moller P. L. Michaelsen K. F. Paerregaard A. (1999). Screening of probiotic activities of forty-seven strains of *Lactobacillus* spp. by *in vitro* techniques and evaluation of the colonization ability of five selected strains in humans. *Appl. Environ. Microbiol.* 65 4949–4956. 10543808 10.1128/aem.65.11.4949-4956.1999 PMC91666

[B132] Jena P. K. Trivedi D. Thakore K. Chaudhary H. Giri S. S. Seshadri S. (2013). Isolation and characterization of probiotic properties of lactobacilli isolated from rat fecal microbiota. *Microbiol. Immunol.* 57 407–416 10.1111/1348-0421.12054 23773019

[B133] Jin J. Zhang B. Guo H. Cui J. Jiang L. Song S. (2012). Mechanism analysis of acid tolerance response of *Bifidobacterium longum* subsp. *longum* BBMN 68 by gene expression profile using RNA-sequencing. *PLoS ONE* 7:e50777 10.1371/journal.pone.0050777 PMC3517610 23236393

[B134] Johnson-Henry K. C. Nadjafi M. Avitzur Y. Mitchell D. J. Ngan B. Y. Galindo-Mata E. (2005). Amelioration of the effects of *Citrobacter rodentium* infection in mice by pretreatment with probiotics. *J. Infect. Dis.* 191 2106–2117 10.1086/430318 15897997

[B135] Joint FAO/WHO Working Group. (2002). *Report on Drafting Guidelines for the Evaluation of Probiotics in Food: Guidelines for the Evaluation of Probiotics in Food.* London, ON: FAO/WHO.

[B136] Jones R. M. Luo L. Ardita C. S. Richardson A. N. Kwon Y. M. Mercante J. W. (2013). Symbiotic lactobacilli stimulate gut epithelial proliferation *via* Nox-mediated generation of reactive oxygen species. *EMBO J.* 32 3017–3028 10.1038/emboj.2013.224 24141879 PMC3844951

[B137] Kang M.-S. Kim B.-G. Chung J. Lee H.-C. Oh J.-S. (2006). Inhibitory effect of *Weissella cibaria* isolates on the production of volatile sulphur compounds. *J. Clin. Periodontol.* 33 226–232 10.1111/j.1600-051X.2006.00893.x 16489950

[B138] Kankainen M. Paulin L. Tynkkynen S. Von Ossowski I. Reunanen J. Partanen P. (2009). Comparative genomic analysis of *Lactobacillus rhamnosus* GG reveals pili containing a human-mucus binding protein. *Proc. Natl. Acad. Sci. U.S.A.* 106 17193–17198 10.1073/pnas.0908876106 19805152 PMC2746127

[B139] Kataoka S. Satoh J. Fujiya H. Toyota T. Suzuki R. Itoh K. (1983). Immunologic aspects of the nonobese diabetic (NOD) mouse. Abnormalities of cellular immunity. *Diabetes* 32 247–253 10.2337/diab.32.3.247 6298042

[B140] Kato I. Endo-Tanaka K. Yokokura T. (1998). Suppressive effects of the oral administration of *Lactobacillus casei* on type II collagen-induced arthritis in DBA/1 mice. *Life Sci.* 63 635–644 10.1016/S0024-3205(98)00315-4 9718093

[B141] Kaur S. Vaishnavi C. Ray P. Kochhar R. Prasad K. K. (2010). Effect of biotherapeutics on cyclosporin-induced *Clostridium difficile* infection in mice. *J. Gastroen. Hepatol.* 25 832–838 10.1111/j.1440-1746.2009.06135.x 20074161

[B142] Kaushik J. K. Kumar A. Duary R. K. Mohanty A. K. Grover S. Batish V. K. (2009). Functional and probiotic attributes of an indigenous isolate of *Lactobacillus plantarum*. *PLoS ONE* 4:e8099 10.1371/journal.pone.0008099 PMC2779496 19956615

[B143] Kechaou N. Chain F. Gratadoux J. J. Blugeon S. Bertho N. Chevalier C. (2013). Identification of one novel candidate probiotic *Lactobacillus plantarum* strain active against influenza virus infection in mice by a large-scale screening. *Appl. Environ. Microbiol.* 79 1491–1499 10.1128/AEM.03075-12 23263960 PMC3591953

[B144] Kikuchi Y. Kunitoh-Asari A. Hayakawa K. Imai S. Kasuya K. Abe K. (2014). Oral administration of *Lactobacillus plantarum* strain AYA enhances IgA secretion and provides survival protection against influenza virus infection in mice. *PLoS ONE* 9:e86416 10.1371/journal.pone.0086416 PMC3899257 24466081

[B145] Kim H. J. Kim Y. J. Lee S. H. Yu J. Jeong S. K. Hong S. J. (2014). Effects of *Lactobacillus rhamnosus* on allergic march model by suppressing Th2, Th17, and TSLP responses via CD4^+^CD25^+^Foxp3^+^ Tregs. *Clin. Immunol.* 153 178–186 10.1016/j.clim.2014.04.008 24769377

[B146] Kim H. S. Gilliland S. E. (1983). *Lactobacillus acidophilus* as a dietary adjunct for milk to aid lactose digestion in humans. *J. Dairy Sci.* 66 959–966 10.3168/jds.S0022-0302(83)81887-6 6409948

[B147] Kim J. F. Jeong H. Yu D. S. Choi S. H. Hur C. G. Park M. S. (2009). Genome sequence of the probiotic bacterium *Bifidobacterium animalis* subsp. *lactis* AD011. *J. Bacteriol.* 191 678–679 10.1128/JB.01515-08 19011029 PMC2620821

[B148] Kim J. Y. Park B. K. Park H. J. Park Y. H. Kim B. O. Pyo S. (2013). Atopic dermatitis-mitigating effects of new *Lactobacillus* strain, *Lactobacillus sakei* probio 65 isolated from Kimchi. *J. Appl. Microbiol.* 115 517–526 10.1111/jam.12229 23607518

[B149] Kim K. H. Park H. S. (2003). Dietary supplementation of conjugated linoleic acid reduces colon tumor incidence in DMH-treated rats by increasing apoptosis with modulation of biomarkers. *Nutrition* 19 772–777 10.1016/S0899-9007(03)00098-4 12921888

[B150] Kinoshita H. Imoto S. Suda Y. Ishida M. Watanabe M. Kawai Y. (2013). Proposal of screening method for intestinal mucus adhesive lactobacilli using the enzymatic activity of glyceraldehyde-3-phosphate dehydrogenase (GAPDH). *Anim. Sci. J.* 84 150–158 10.1111/j.1740-0929.2012.01054.x 23384357

[B151] Kirjavainen P. V. Ouwehand A. C. Isolauri E. Salminen S. J. (1998). The ability of probiotic bacteria to bind to human intestinal mucus. *FEMS Microbiol. Lett.* 167 185–189 10.1111/j.1574-6968.1998.tb13226.x 9809419

[B152] Kiyohara M. Nakatomi T. Kurihara S. Fushinobu S. Suzuki H. Tanaka T. (2012). α-*N*-acetylgalactosaminidase from infant-associated bifidobacteria belonging to novel glycoside hydrolase family 129 is implicated in alternative mucin degradation pathway. *J. Biol. Chem.* 287 693–700 10.1074/jbc.M111.277384 22090027 PMC3249124

[B153] Knight A. (2007). Animal experiments scrutinised: systematic reviews demonstrate poor human clinical and toxicological utility. *ALTEX* 24 320–325. 18288428 10.14573/altex.2007.4.320

[B154] Konstantinov S. R. Smidt H. De Vos W. M. Bruijns S. C. Singh S. K. Valence F. (2008). S layer protein A of *Lactobacillus acidophilus* NCFM regulates immature dendritic cell and T cell functions. *Proc. Natl. Acad. Sci. U.S.A.* 105 19474–19479 10.1073/pnas.0810305105 19047644 PMC2592362

[B155] Koponen J. Laakso K. Koskenniemi K. Kankainen M. Savijoki K. Nyman T. A. (2012). Effect of acid stress on protein expression and phosphorylation in *Lactobacillus rhamnosus* GG. *J. Proteomics* 75 1357–1374 10.1016/j.jprot.2011.11.009 22119544

[B156] Koskenniemi K. Laakso K. Koponen J. Kankainen M. Greco D. Auvinen P. (2011). Proteomics and transcriptomics characterization of bile stress response in probiotic *Lactobacillus rhamnosus* GG. *Mol. Cell. Proteomics* 10 M110. 002741. 10.1074/mcp.M110.002741 PMC3033674 21078892

[B157] Kostic A. D. Howitt M. R. Garrett W. S. (2013). Exploring host-microbiota interactions in animal models and humans. *Genes Dev.* 27 701–718 10.1101/gad.212522.112 23592793 PMC3639412

[B158] Krasse P. Carlsson B. Dahl C. Paulsson A. Nilsson A. Sinkiewicz G. (2006). Decreased gum bleeding and reduced gingivitis by the probiotic *Lactobacillus reuteri*. Swed. *Dent. J.* 30 55–60. 16878680

[B159] Kullisaar T. Zilmer M. Mikelsaar M. Vihalemm T. Annuk H. Kairane C. (2002). Two antioxidative lactobacilli strains as promising probiotics. *Int. J. Food Microbiol.* 72 215–224 10.1016/S0168-1605(01)00674-2 11845820

[B160] Kumar M. Kumar A. Nagpal R. Mohania D. Behare P. Verma V. (2010). Cancer-preventing attributes of probiotics: an update. *Int. J. Food Sci. Nutr.* 61 473–496 10.3109/09637480903455971 20187714

[B161] Kwon H. K. Kim G. C. Kim Y. Hwang W. Jash A. Sahoo A. (2013). Amelioration of experimental autoimmune encephalomyelitis by probiotic mixture is mediated by a shift in T helper cell immune response. *Clin. Immunol.* 146 217–227 10.1016/j.clim.2013.01.001 23416238

[B162] Lahtinen S. J. Jalonen L. Ouwehand A. C. Salminen S. J. (2007). Specific *Bifidobacterium* strains isolated from elderly subjects inhibit growth of *Staphylococcus aureus*. *Int. J. Food Microbiol.* 117 125–128 10.1016/j.ijfoodmicro.2007.02.023 17462772

[B163] Lahteinen T. Malinen E. Koort J. M. Mertaniemi-Hannus U. Hankimo T. Karikoski N. (2010). Probiotic properties of *Lactobacillus* isolates originating from porcine intestine and feces. *Anaerobe* 16 293–300 10.1016/j.anaerobe.2009.08.002 19695336

[B164] Laparra J. M. Sanz Y. (2009). Comparison of *in vitro* models to study bacterial adhesion to the intestinal epithelium. *Lett. Appl. Microbiol.* 49 695–701 10.1111/j.1472-765X.2009.02729.x 19843211

[B165] Lavermicocca P. Valerio F. Lonigro S. L. Di Leo A. Visconti A. (2008). Antagonistic activity of potential probiotic lactobacilli against the ureolytic pathogen *Yersinia enterocolitica*. *Curr. Microbiol.* 56 175–181 10.1007/s00284-007-9069-5 18074177

[B166] Lebeer S. Claes I. Tytgat H. L. Verhoeven T. L. Marien E. Von Ossowski I. (2012). Functional analysis of *Lactobacillus rhamnosus* GG pili in relation to adhesion and immunomodulatory interactions with intestinal epithelial cells. *Appl. Environ. Microbiol.* 78 185–193 10.1128/AEM.06192-11 22020518 PMC3255643

[B167] Lebeer S. Claes I. J. J. Verhoeven T. L. A. Vanderleyden J. De Keersmaecker S. C. J. (2011). Exopolysaccharides of *Lactobacillus rhamnosus* GG form a protective shield against innate immune factors in the intestine. *Microb. Biotechnol.* 4 368–374 10.1111/j.1751-7915.2010.00199.x 21375696 PMC3818995

[B168] Lebeer S. Vanderleyden J. De Keersmaecker S. C. (2008). Genes and molecules of lactobacilli supporting probiotic action. *Microbiol. Mol. Biol. R.* 72 728–764 10.1128/MMBR.00017 PMC2593565 19052326

[B169] Lee H. Y. Park J. H. Seok S. H. Baek M. W. Kim D. J. Lee K. E. (2006). Human originated bacteria, *Lactobacillus rhamnosus* PL60, produce conjugated linoleic acid and show anti-obesity effects in diet-induced obese mice. *Biochim. Biophys. Acta* 1761 736–744 10.1016/j.bbalip.2006.05.007 16807088

[B170] Lee J. Kim Y. Yun H. S. Kim J. G. Oh S. Kim S. H. (2010). Genetic and proteomic analysis of factors affecting serum cholesterol reduction by *Lactobacillus acidophilus* A4. *Appl. Environ. Microbiol.* 76 4829–4835 10.1128/AEM.02892 20495044 PMC2901756

[B171] Lee J. W. Shin J. G. Kim E. H. Kang H. E. Yim I. B. Kim J. Y. (2004). Immunomodulatory and antitumor effects *in vivo* by the cytoplasmic fraction of *Lactobacillus casei* and *Bifidobacterium longum*. *J. Vet. Sci.* 5 41–48. 15028884

[B172] Leist M. Hartung T. (2013). Inflammatory findings on species extrapolations: humans are definitely no 70-kg mice. *Arch. Toxicol.* 87 563–567 10.1007/s00204-013-1038-0 23503654 PMC3604596

[B173] Le Leu R. K. Brown I. L. Hu Y. Bird A. R. Jackson M. Esterman A. (2005). A synbiotic combination of resistant starch and *Bifidobacterium lactis* facilitates apoptotic deletion of carcinogen-damaged cells in rat colon. *J. Nutr.* 135 996–1001. 15867271 10.1093/jn/135.5.996

[B174] Le Marechal C. Peton V. Ple C. Vroland C. Jardin J. Briard-Bion V. (2014). Surface proteins of *Propionibacterium freudenreichii* are involved in its anti-inflammatory properties. *J. Proteomics.* 113 447–461 10.1016/j.jprot.2014.07.018 25150945

[B175] Le Roy T. Llopis M. Lepage P. Bruneau A. Rabot S. Bevilacqua C. (2013). Intestinal microbiota determines development of non-alcoholic fatty liver disease in mice. *Gut* 62 1787–1794 10.1136/gutjnl-2012-303816 23197411

[B176] Lessman C. A. (2011). The developing zebrafish (*Danio rerio*): a vertebrate model for high-throughput screening of chemical libraries. *Birth Defects Res. C Embryo Today* 93 268–280 10.1002/bdrc.20212 21932435

[B177] Ley R. E. Backhed F. Turnbaugh P. Lozupone C. A. Knight R. D. Gordon J. I. (2005). Obesity alters gut microbial ecology. *Proc. Natl. Acad. Sci. U.S.A.* 102 11070–11075 10.1073/pnas.0504978102 16033867 PMC1176910

[B178] Lieschke G. J. Currie P. D. (2007). Animal models of human disease: zebrafish swim into view. *Nat. Rev. Genet.* 8 353–367 10.1038/nrg2091 17440532

[B179] Lye H. S. Rusul G. Liong M. T. (2010). Removal of cholesterol by lactobacilli via incorporation and conversion to coprostanol. *J. Dairy Sci.* 93 1383–1392 10.3168/jds.2009-2574 20338415

[B180] Macho Fernandez E. Pot B. Grangette C. (2011). Beneficial effect of probiotics in IBD: are peptidogycan and *NOD2* the molecular key effectors? *Gut Microbes* 2 280–286 10.4161/gmic.2.5.18255 22067939

[B181] Mack D. R. Ahrne S. Hyde L. Wei S. Hollingsworth M. A. (2003). Extracellular MUC3 mucin secretion follows adherence of *Lactobacillus* strains to intestinal epithelial cells *in vitro*. *Gut* 52 827–833 10.1136/gut.52.6.827 12740338 PMC1773687

[B182] Mackenzie D. A. Jeffers F. Parker M. L. Vibert-Vallet A. Bongaerts R. J. Roos S. (2010). Strain-specific diversity of mucus-binding proteins in the adhesion and aggregation properties of *Lactobacillus reuteri*. *Microbiology* 156 3368–3378 10.1099/mic.0.043265-0 20847011

[B183] Mainville I. Arcand Y. Farnworth E. R. (2005). A dynamic model that simulates the human upper gastrointestinal tract for the study of probiotics. *Int. J. Food Microbiol.* 99 287–296 10.1016/j.ijfoodmicro.2004.08.020 15808363

[B184] Malik S. Petrova M. I. Claes I. J. Verhoeven T. L. Busschaert P. Vaneechoutte M. (2013). The highly autoaggregative and adhesive phenotype of the vaginal *Lactobacillus plantarum* strain CMPG5300 is sortase dependent. *Appl. Environ. Microbiol.* 79 4576–4585 10.1128/AEM.00926-13 23709503 PMC3719525

[B185] Mandlik A. Swierczynski A. Das A. Ton-That H. (2008). Pili in Gram-positive bacteria: assembly, involvement in colonization and biofilm development. *Trends Microbiol.* 16 33–40 10.1016/j.tim.2007.10.010 18083568 PMC2841691

[B186] Mariadason J. M. Catto-Smith A. Gibson P. R. (1999). Modulation of distal colonic epithelial barrier function by dietary fibre in normal rats. *Gut* 44 394–399 10.1136/gut.44.3.394 10026327 PMC1727405

[B187] Marteau P. Minekus M. Havenaar R. Huis In’t Veld J. H. J. (1997). Survival of lactic acid bacteria in a dynamic model of the stomach and small intestine: validation and the effects of bile. *J. Dairy Sci.* 80 1031–1037 10.3168/jds.S0022-0302(97)76027-2 9201571

[B188] Martin F. P. Wang Y. Sprenger N. Yap I. K. Rezzi S. Ramadan Z. (2008). Top-down systems biology integration of conditional prebiotic modulated transgenomic interactions in a humanized microbiome mouse model. *Mol. Syst. Biol.* 4 205 10.1038/msb.2008.40 PMC2516362 18628745

[B189] Matos R. C. Leulier F. (2014). Lactobacilli-host mutualism: learning on the fly. *Microb. Cell Fact.* 13(Suppl. 1), S6 10.1186/1475-2859-13-S1-S6 PMC4155823 25186369

[B190] Matsumoto S. Hara T. Nagaoka M. Mike A. Mitsuyama K. Sako T. (2009). A component of polysaccharide peptidoglycan complex on *Lactobacillus* induced an improvement of murine model of inflammatory bowel disease and colitis-associated cancer. *Immunology* 128 e170–e180 10.1111/j.1365-2567.2008.02942.x 19740306 PMC2753921

[B191] Matsuzaki T. Yokokura T. Azuma I. (1985). Anti-tumour activity of *Lactobacillus casei* on Lewis lung carcinoma and line-10 hepatoma in syngeneic mice and guinea pigs. *Cancer Immunol. Immunother.* 20 18–22 10.1007/BF00199768 3933816 PMC11038615

[B192] Matsuzaki T. Yokokura T. Mutai M. (1988). Antitumor effect of intrapleural administration of *Lactobacillus casei* in mice. *Cancer Immunol. Immunother.* 26 209–214 10.1007/BF00199931 3133110 PMC11038425

[B193] McKay D. M. Philpott D. J. Perdue M. H. (1997). Review article: *in vitro* models in inflammatory bowel disease research-a critical review. *Aliment. Pharmacol. Ther.* 11(Suppl. 3), 70–80 10.1111/j.1365-2036.1997.tb00811.x 9467981

[B194] Meijerink M. Van Hemert S. Taverne N. Wels M. De Vos P. Bron P. A. (2010). Identification of genetic loci in *Lactobacillus plantarum* that modulate the immune response of dendritic cells using comparative genome hybridization. *PLoS ONE* 5:e10632 10.1371/journal.pone.0010632 PMC2869364 20498715

[B195] Mennigen R. Bruewer M. (2009). Effect of probiotics on intestinal barrier function. *Ann. N. Y. Acad. Sci.* 1165 183–189 10.1111/j.1749-6632.2009.04059.x 19538305

[B196] Miettinen M. Matikainen S. Vuopio-Varkila J. Pirhonen J. Varkila K. Kurimoto M. (1998). Lactobacilli and Streptococci induce interleukin-12 (IL-12), IL-18, and gamma interferon production in human peripheral blood mononuclear cells. *Infect. Immun.* 66 6058–6062. 9826398 10.1128/iai.66.12.6058-6062.1998 PMC108774

[B197] Minekus M. Smeets-Peeters M. Bernalier A. Marol-Bonnin S. Havenaar R. Marteau P. (1999). A computer-controlled system to simulate conditions of the large intestine with peristaltic mixing, water absorption and absorption of fermentation products. *Appl. Microbiol. Biotechnol.* 53 108–114 10.1007/s002530051622 10645630

[B198] Miquel S. Martín R. Rossi O. Bermúdez-Humarán L. G. Chatel J. M. Sokol H. (2013). *Faecalibacterium prausnitzii* and human intestinal health. *Curr. Opin. Microbiol.* 16 255–261 10.1016/j.mib.2013.06.003 23831042

[B199] Mitsuma T. Odajima H. Momiyama Z. Watanabe K. Masuguchi M. Sekine T. (2008). Enhancement of gene expression by a peptide p(CHWPR) produced by *Bifidobacterium lactis* BB-12. *Microbiol. Immunol.* 52 144–155 10.1111/j.1348-0421.2008.00022.x 18402596

[B200] Molly K. Woestyne M. V. Smet I. D. (1994). Validation of the simulator of the human intestinal microbial ecosystem (SHIME) reactor using microorganism-associated activities. *Microb. Ecol. Health Dis.* 7 191–200 10.3109/08910609409141354

[B201] Moorehead R. J. Hoper M. Mckelvey S. T. (1987). Assessment of ornithine decarboxylase activity in rectal mucosa as a marker for colorectal adenomas and carcinomas. *Br. J. Surg.* 74 364–365 10.1002/bjs.1800740513 3594125

[B202] Moser A. R. Luongo C. Gould K. A. Mcneley M. K. Shoemaker A. R. Dove W. F. (1995). *Apc^Min^*: a mouse model for intestinal and mammary tumorigenesis. *Eur. J. Cancer* 31A, 1061–1064 10.1016/0959-8049(95)00181-H 7576992

[B203] Moslehi-Jenabian S. Gori K. Jespersen L. (2009). AI-2 signalling is induced by acidic shock in probiotic strains of *Lactobacillus* spp. *Int. J. Food Microbiol.* 135 295–302 10.1016/j.ijfoodmicro.2009.08.011 19748697

[B204] Moslehi-Jenabian S. Vogensen F. K. Jespersen L. (2011). The quorum sensing *luxS* gene is induced in *Lactobacillus acidophilus* NCFM in response to *Listeria monocytogenes*. *Int. J. Food Microbiol.* 149 269–273 10.1016/j.ijfoodmicro.2011.06.011 21784546

[B205] Muller J. A. Ross R. P. Fitzgerald G. F. Stanton C. (2009). “Manufacture of probiotic bacteria,” in *Prebiotics and Probiotics Science and Technology*, eds Charalampopoulos D. Rastall R. (New York: Springer), 725–759 10.1007/978-0-387-79058-9_18

[B206] Munoz-Provencio D. Rodriguez-Diaz J. Collado M. C. Langella P. Bermudez-Humaran L. G. Monedero V. (2012). Functional analysis of the *Lactobacillus casei* BL23 sortases. *Appl. Environ. Microbiol.* 78 8684–8693 10.1128/AEM.02287-12 23042174 PMC3502915

[B207] Nadal I. Santacruz A. Marcos A. Warnberg J. Garagorri J. M. Moreno L. A. (2009). Shifts in clostridia, bacteroides and immunoglobulin-coating fecal bacteria associated with weight loss in obese adolescents. *Int. J. Obes. (Lond.)* 33 758–767 10.1038/ijo.2008.260 19050675

[B208] Nicholson J. K. Holmes E. Wilson I. D. (2005). Gut microorganisms, mammalian metabolism and personalized health care. *Nat. Rev. Microbiol.* 3 431–438 10.1038/nrmicro1152 15821725

[B209] Nybom S. M. Salminen S. J. Meriluoto J. A. (2008). Specific strains of probiotic bacteria are efficient in removal of several different cyanobacterial toxins from solution. *Toxicon* 52 214–220 10.1016/j.toxicon.2008.04.169 18639912

[B210] Oakey H. J. Harty D. W. Knox K. W. (1995). Enzyme production by lactobacilli and the potential link with infective endocarditis. *J. Appl. Bacteriol.* 78 142–148 10.1111/j.1365-2672.1995.tb02834.x 7698950

[B211] Oehlers S. H. Flores M. V. Hall C. J. Swift S. Crosier K. E. Crosier P. S. (2011). The inflammatory bowel disease (IBD) susceptibility genes *NOD1* and *NOD2* have conserved anti-bacterial roles in zebrafish. *Dis. Model. Mech.* 4 832–841 10.1242/dmm.006122 21729873 PMC3209652

[B212] O’Mahony L. Feeney M. O’halloran S. Murphy L. Kiely B. Fitzgibbon J. (2001). Probiotic impact on microbial flora, inflammation and tumour development in IL-10 knockout mice. *Aliment. Pharmacol. Ther.* 15 1219–1225 10.1046/j.1365-2036.2001.01027.x 11472326

[B213] Orrhage K. M. Annas A. Nord C. E. Brittebo E. B. Rafter J. J. (2002). Effects of lactic acid bacteria on the uptake and distribution of the food mutagen Trp-P-2 in mice. *Scand. J. Gastroenterol.* 37 215–221 10.1080/003655202753416902 11843060

[B214] Ouwehand A. C. Kirjavainen P. V. Grönlund M. M. Isolauri E. Salminen S. J. (1999). Adhesion of probiotic micro-organisms to intestinal mucus. *Int. Dairy J.* 9 623–630 10.1016/S0958-6946(99)00132-6

[B215] Pan X. Yang Y. Zhang J.-R. (2014). Molecular basis of host specificity in human pathogenic bacteria. *Emerg. Microbes Infect.* 3:e23 10.1038/emi.2014.23 PMC3974339 26038515

[B216] Papadimitriou C. G. Vafopoulou-Mastrojiannaki A. Silva S. V. Gomes A.-M. Malcata F. X. Alichanidis E. (2007). Identification of peptides in traditional and probiotic sheep milk yoghurt with angiotensin I-converting enzyme (ACE)-inhibitory activity. *Food Chem.* 105 647–656 10.1016/j.foodchem.2007.04.028

[B217] Park M. R. Yun H. S. Son S. J. Oh S. Kim Y. (2014). Short communication: development of a direct *in vivo* screening model to identify potential probiotic bacteria using *Caenorhabditis elegans*. *J. Dairy Sci.* 97 6828–6834 10.3168/jds.2014-8561 25200770

[B218] Parkes G. C. Sanderson J. D. Whelan K. (2009). The mechanisms and efficacy of probiotics in the prevention of *Clostridium difficile*-associated diarrhoea. *Lancet Infect. Dis.* 9 237–244 10.1016/S1473-3099(09)70059-3 19324296

[B219] Perea Velez M. Verhoeven T. L. Draing C. Von Aulock S. Pfitzenmaier M. Geyer A. (2007). Functional analysis of D-alanylation of lipoteichoic acid in the probiotic strain *Lactobacillus rhamnosus* GG. *Appl. Environ. Microbiol.* 73 3595–3604 10.1128/AEM.02083 17434999 PMC1932685

[B220] Pisano M. B. Viale S. Conti S. Fadda M. E. Deplano M. Melis M. P. (2014). Preliminary evaluation of probiotic properties of *Lactobacillus* strains isolated from Sardinian dairy products. *Biomed Res. Int.* 2014:286390 10.1155/2014/286390 PMC4099116 25054135

[B221] Pokusaeva K. Fitzgerald G. F. Van Sinderen D. (2011). Carbohydrate metabolism in bifidobacteria. *Genes Nutr.* 6 285–306 10.1007/s12263-010-0206-6 21484167 PMC3145055

[B222] Pompei A. Cordisco L. Amaretti A. Zanoni S. Matteuzzi D. Rossi M. (2007). Folate production by bifidobacteria as a potential probiotic property. *Appl. Environ. Microbiol.* 73 179–185 10.1128/AEM.01763 17071792 PMC1797147

[B223] Pool-Zobel B. L. Neudecker C. Domizlaff I. Ji S. Schillinger U. Rumney C. (1996). *Lactobacillus*- and *Bifidobacterium*-mediated antigenotoxicity in the colon of rats. *Nutr. Cancer* 26 365–380 10.1080/01635589609514492 8910918

[B224] Price C. E. Reid S. J. Driessen A. J. Abratt V. R. (2006). The *Bifidobacterium longum* NCIMB 702259^T^ *ctr* gene codes for a novel cholate transporter. *Appl. Environ. Microbiol.* 72 923–926 10.1128/AEM.72.1.923-926.2006 16391136 PMC1352250

[B225] Qin J. Li R. Raes J. Arumugam M. Burgdorf K. S. Manichanh C. (2010). A human gut microbial gene catalogue established by metagenomic sequencing. *Nature* 464 59–65 10.1038/nature08821 20203603 PMC3779803

[B226] Rastmanesh R. (2011). High polyphenol, low probiotic diet for weight loss because of intestinal microbiota interaction. *Chem. Biol. Interact.* 189 1–8 10.1016/j.cbi.2010.10.002 20955691

[B227] Ravel J. Blaser M. Braun J. Brown E. Bushman F. Chang E. (2014). Human microbiome science: vision for the future, Bethesda, MD, July 24 to 26, 2013. *Microbiome* 2:16 10.1186/2049-2618-2-16

[B228] Resta-Lenert S. Barrett K. E. (2003). Live probiotics protect intestinal epithelial cells from the effects of infection with enteroinvasive *Escherichia coli* (EIEC). *Gut* 52 988–997 10.1136/gut.52.7.988 12801956 PMC1773702

[B229] Richmond J. (2000). The 3Rs – past, present and future. *Scand. J. Lab. Anim. Sci.* 27 84–92.

[B230] Rieu A. Aoudia N. Jego G. Chluba J. Yousfi N. Briandet R. (2014). The biofilm mode of life boosts the anti-inflammatory properties of *Lactobacillus*. *Cell. Microbiol.* 16 1836–1853 10.1111/cmi.12331 25052472

[B231] Rijkers G. T. Bengmark S. Enck P. Haller D. Herz U. Kalliomaki M. (2010). Guidance for substantiating the evidence for beneficial effects of probiotics: current status and recommendations for future research. *J. Nutr.* 140 671S–676S 10.3945/jn.109.113779 20130080

[B232] Rousseaux C. Thuru X. Gelot A. Barnich N. Neut C. Dubuquoy L. (2007). *Lactobacillus acidophilus* modulates intestinal pain and induces opioid and cannabinoid receptors. *Nat. Med.* 13 35–37 10.1038/nm1521 17159985

[B233] Ruas-Madiedo P. Gueimonde M. Fernandez-Garcia M. De Los Reyes-Gavilan C. G. Margolles A. (2008). Mucin degradation by *Bifidobacterium* strains isolated from the human intestinal microbiota. *Appl. Environ. Microbiol.* 74 1936–1940 10.1128/AEM.02509-07 18223105 PMC2268317

[B234] Ruiz L. Zomer A. O’Connell-Motherway M. Van Sinderen D. Margolles A. (2012). Discovering novel bile protection systems in *Bifidobacterium breve* UCC2003 through functional genomics. *Appl. Environ. Microbiol.* 78 1123–1131 10.1128/AEM.06060-11 22156415 PMC3273026

[B235] Russel W. M. S. Burch R. L. (1959). *The Principles of Humane Experimental Technique.* London: Universities Federation for Animal Welfare, Wheathampstead.

[B236] Ryan S. M. Fitzgerald G. F. Van Sinderen D. (2005). Transcriptional regulation and characterization of a novel β-fructofuranosidase-encoding gene from *Bifidobacterium breve* UCC2003. *Appl. Environ. Microbiol.* 71 3475–3482 10.1128/AEM.71.7.3475-3482.2005 16000751 PMC1169055

[B237] Sakata T. Kojima T. Fujieda M. Takahashi M. Michibata T. (2003). Influences of probiotic bacteria on organic acid production by pig caecal bacteria *in vitro*. *Proc. Nutr. Soc.* 62 73–80 10.1079/PNS2002211 12740061

[B238] Salminen S. Ouwehand A. Benno Y. Lee Y. K. (1999). Probiotics: how should they be defined? *Trends Food Sci. Technol*. 10 107–110 10.1016/S0924-2244(99)00027-8

[B239] Sanchez B. Champomier-Verges M. C. Anglade P. Baraige F. De Los Reyes-Gavilan C. G. Margolles A. (2005). Proteomic analysis of global changes in protein expression during bile salt exposure of *Bifidobacterium longum* NCIMB 8809. *J. Bacteriol.* 187 5799–5808 10.1128/JB.187.16.5799-5808.2005 16077128 PMC1196055

[B240] Sanchez B. Champomier-Verges M. C. Stuer-Lauridsen B. Ruas-Madiedo P. Anglade P. Baraige F. (2007). Adaptation and response of *Bifidobacterium animalis* subsp. *lactis* to bile: a proteomic and physiological approach. *Appl. Environ. Microbiol.* 73 6757–6767 10.1128/AEM.00637-07 17827318 PMC2074956

[B241] Sanchez B. De Los Reyes-Gavilan C. G. Margolles A. (2006). The F_1_F_0_-ATPase of *Bifidobacterium animalis* is involved in bile tolerance. *Environ. Microbiol.* 8 1825–1833 10.1111/j.1462-2920.2006.01067.x 16958763

[B242] Sanchez B. Ruiz L. De Los Reyes-Gavilan C. G. Margolles A. (2008). Proteomics of stress response in *Bifidobacterium*. *Front. Biosci.* 13:6905. 10.2741/3198 18508704

[B243] Sanders M. E. Akkermans L. M. Haller D. Hammerman C. Heimbach J. Hormannsperger G. (2010). Safety assessment of probiotics for human use. *Gut Microbes* 1 164–185 10.4161/gmic.1.3.12127 21327023 PMC3023597

[B244] Saulnier D. M. Santos F. Roos S. Mistretta T. A. Spinler J. K. Molenaar D. (2011). Exploring metabolic pathway reconstruction and genome-wide expression profiling in *Lactobacillus reuteri* to define functional probiotic features. *PLoS ONE* 6:e18783 10.1371/journal.pone.0018783 PMC3084715 21559529

[B245] Scaldaferri F. Gerardi V. Lopetuso L. R. Del Zompo F. Mangiola F. Boskoski I. (2013). Gut microbial flora, prebiotics, and probiotics in IBD: their current usage and utility. *Biomed. Res. Int.* 2013 435268 10.1155/2013/435268 PMC3749555 23991417

[B246] Schlee M. Wehkamp J. Altenhoefer A. Oelschlaeger T. A. Stange E. F. Fellermann K. (2007). Induction of human β-defensin 2 by the probiotic *Escherichia coli* Nissle 1917 is mediated through flagellin. *Infect. Immun.* 75 2399–2407 10.1128/IAI.01563 17283097 PMC1865783

[B247] Sekine K. Toida T. Saito M. Kuboyama M. Kawashima T. Hashimoto Y. (1985). A new morphologically characterized cell wall preparation (whole peptidoglycan) from *Bifidobacterium infantis* with a higher efficacy on the regression of an established tumor in mice. *Cancer Res.* 45 1300–1307. 3971375

[B248] Sela D. A. Chapman J. Adeuya A. Kim J. H. Chen F. Whitehead T. R. (2008). The genome sequence of *Bifidobacterium longum* subsp. *infantis* reveals adaptations for milk utilization within the infant microbiome. *Proc. Natl. Acad. Sci. U.S.A.* 105 18964–18969 10.1073/pnas.0809584105 19033196 PMC2596198

[B249] Seok J. Warren H. S. Cuenca A. G. Mindrinos M. N. Baker H. V. Xu W. (2013). Genomic responses in mouse models poorly mimic human inflammatory diseases. *Proc. Natl. Acad. Sci. U.S.A.* 110 3507–3512 10.1073/pnas.1222878110 23401516 PMC3587220

[B250] Shanks N. Greek R. Greek J. (2009). Are animal models predictive for humans? *Philos. Ethics Humanit. Med*. 4 2 10.1186/1747-5341-4-2 PMC2642860 19146696

[B251] Shimada Y. Watanabe Y. Wakinaka T. Funeno Y. Kubota M. Chaiwangsri T. (2014). α-*N*-Acetylglucosaminidase from *Bifidobacterium bifidum* specifically hydrolyzes α-linked *N*-acetylglucosamine at nonreducing terminus of *O*-glycan on gastric mucin. *Appl. Microbiol. Biotechnol.* 10.1007/s00253-014-6201-x [Epub ahead of print]. 25381911

[B252] Siniscalco D. Antonucci N. (2013). Involvement of dietary bioactive proteins and peptides in autism spectrum disorders. *Curr. Protein Pept. Sci.* 14 674–679 10.2174/1389203711209070602 24106964

[B253] Smet I. D. Hoorde L. V. Saeyer N. D. Woestyne M. V. Verstraete W. (1994). *In vitro* study of bile salt hydrolase (BSH) activity of BSH isogenic *Lactobacillus plantarum* 80 strains and estimation of cholesterol lowering through enhanced BSH activity. *Microb. Ecol. Health Dis.* 7 315–329.

[B254] Smits H. H. Engering A. Van Der Kleij D. De Jong E. C. Schipper K. Van Capel T. M. (2005). Selective probiotic bacteria induce IL-10-producing regulatory T cells *in vitro* by modulating dendritic cell function through dendritic cell-specific intercellular adhesion molecule 3-grabbing nonintegrin. *J. Allergy Clin. Immunol.* 115 1260–1267 10.1016/j.jaci.2005.03.036 15940144

[B255] Sodhi C. P. Neal M. D. Siggers R. Sho S. Ma C. Branca M. F. (2012). Intestinal epithelial Toll-like receptor 4 regulates goblet cell development and is required for necrotizing enterocolitis in mice. *Gastroenterology* 143 708–718, e701–e705 10.1053/j.gastro.2012.05.053 22796522 PMC3584415

[B256] Songisepp E. Kals J. Kullisaar T. Mändar R. Hütt P. Zilmer M. (2005). Evaluation of the functional efficacy of an antioxidative probiotic in healthy volunteers. *Nutr. J.* 4 22–22 10.1186/1475-2891-4-22 16080791 PMC1198254

[B257] Steinberg R. S. Silva L. C. Souza T. C. Lima M. T. De Oliveira N. L. Vieira L. Q. (2014). Safety and protective effectiveness of two strains of *Lactobacillus* with probiotic features in an experimental model of salmonellosis. *Int. J. Environ. Res. Public Health* 11 8755–8776 10.3390/ijerph110908755 25162711 PMC4198989

[B258] Strus M. Kucharska A. Kukla G. Brzychczy-Wloch M. Maresz K. Heczko P. B. (2005). The *in vitro* activity of vaginal *Lactobacillus* with probiotic properties against *Candida*. *Infect. Dis. Obstet. Gynecol.* 13 69–75 10.1080/10647440400028136 16011996 PMC1784560

[B259] Sturme M. H. Nakayama J. Molenaar D. Murakami Y. Kunugi R. Fujii T. (2005). An *agr*-like two-component regulatory system in *Lactobacillus plantarum* is involved in production of a novel cyclic peptide and regulation of adherence. *J. Bacteriol.* 187 5224–5235 10.1128/JB.187.15.5224-5235.2005 16030216 PMC1196011

[B260] Suardi E. Crippa F. D’arminio Monforte A. (2013). Probiotics in the prevention of antibiotic-associated diarrhea in adults. *Int. J. Probiotics Prebiotics* 8 41–44.

[B261] Sun X. Wang H. Zhang Y. Chen K. Davis B. Feng H. (2011). Mouse relapse model of *Clostridium difficile* infection. *Infect. Immun.* 79 2856–2864 10.1128/IAI.01336-10 21576341 PMC3191975

[B262] Tagg J. R. McGiven A. R. (1971). Assay system for bacteriocins. *Appl. Microbiol.* 21 943. 10.1128/am.21.5.943-943.1971 PMC377313 4930039

[B263] Tan Q. Xu H. Aguilar Z. P. Peng S. Dong S. Wang B. (2013). Safety assessment and probiotic evaluation of *Enterococcus faecium* YF5 isolated from sourdough. *J. Food Sci.* 78 M587–M593 10.1111/1750-3841.12079 23488799

[B264] Tassell M. L. V. Miller M. J. (2011). *Lactobacillus* adhesion to mucus. *Nutrients* 3 613–636 10.3390/nu3050613 22254114 PMC3257693

[B265] Tomaro-Duchesneau C. Jones M. L. Shah D. Jain P. Saha S. Prakash S. (2014). Cholesterol assimilation by *Lactobacillus* probiotic bacteria: an *in vitro* investigation. *Biomed Res. Int.* 2014 380316 10.1155/2014/380316 PMC4176637 25295259

[B266] Topping D. L. Clifton P. M. (2001). Short-chain fatty acids and human colonic function: roles of resistant starch and nonstarch polysaccharides. *Physiol. Rev.* 81 1031–1064. 11427691 10.1152/physrev.2001.81.3.1031

[B267] Turnbaugh P. J. Backhed F. Fulton L. Gordon J. I. (2008). Diet-induced obesity is linked to marked but reversible alterations in the mouse distal gut microbiome. *Cell Host Microbe* 3 213–223 10.1016/j.chom.2008.02.015 18407065 PMC3687783

[B268] Turnbaugh P. J. Ley R. E. Mahowald M. A. Magrini V. Mardis E. R. Gordon J. I. (2006). An obesity-associated gut microbiome with increased capacity for energy harvest. *Nature* 444 1027–1031 10.1038/nature05414 17183312

[B269] Turpin W. Humblot C. Noordine M. L. Thomas M. Guyot J. P. (2012). *Lactobacillaceae* and cell adhesion: genomic and functional screening. *PLoS ONE* 7:e38034 10.1371/journal.pone.0038034 PMC3364998 22675431

[B270] Turroni F. Bottacini F. Foroni E. Mulder I. Kim J. H. Zomer A. (2010a). Genome analysis of *Bifidobacterium bifidum* PRL2010 reveals metabolic pathways for host-derived glycan foraging. *Proc. Natl. Acad. Sci. U.S.A.* 107 19514–19519 10.1073/pnas.1011100107 20974960 PMC2984195

[B271] Turroni F. Foroni E. O’connell Motherway M. Bottacini F. Giubellini V. Zomer A. (2010b). Characterization of the serpin-encoding gene of *Bifidobacterium breve* 210B. *Appl. Environ. Microbiol.* 76 3206–3219 10.1128/AEM.02938 20348296 PMC2869134

[B272] Turroni F. Milani C. Van Sinderen D. Ventura M. (2011). Genetic strategies for mucin metabolism in *Bifidobacterium bifidum* PRL2010: an example of possible human-microbe co-evolution. *Gut Microbes* 2 183–189 10.4161/gmic.2.3.16105 21804355

[B273] Turroni F. Serafini F. Foroni E. Duranti S. O’connell Motherway M. Taverniti V. (2013). Role of sortase-dependent pili of *Bifidobacterium bifidum* PRL2010 in modulating bacterium-host interactions. *Proc. Natl. Acad. Sci. U.S.A.* 110 11151–11156 10.1073/pnas.1303897110 23776216 PMC3703987

[B274] Uccello M. Malaguarnera G. Basile F. D’agata V. Malaguarnera M. Bertino G. (2012). Potential role of probiotics on colorectal cancer prevention. *BMC Surg.* 12(Suppl. 1):S35 10.1186/1471-2482-12-S1-S35 PMC3499195 23173670

[B275] Upadrasta A. Stanton C. Hill C. Fitzgerald G. Ross R. P. (2011). “Improving the stress tolerance of probiotic cultures: recent trends and future directions,” in *Stress Responses of Lactic Acid Bacteria*, eds Tsakalidou E. Papadimitriou K. (New York: Springer), 395–438.

[B276] van Baarlen P. Troost F. J. Van Hemert S. Van Der Meer C. De Vos W. M. De Groot P. J. (2009). Differential NF-κB pathways induction by *Lactobacillus plantarum* in the duodenum of healthy humans correlating with immune tolerance. *Proc. Natl. Acad. Sci. U.S.A.* 106 2371–2376 10.1073/pnas.0809919106 19190178 PMC2650163

[B277] van Bokhorst-van de Veen H. Lee I. C. Marco M. L. Wels M. Bron P. A. Kleerebezem M. (2012). Modulation of *Lactobacillus plantarum* gastrointestinal robustness by fermentation conditions enables identification of bacterial robustness markers. *PLoS ONE* 7:e39053 10.1371/journal.pone.0039053 PMC3389004 22802934

[B278] Van den Abbeele P. Roos S. Eeckhaut V. Mackenzie D. A. Derde M. Verstraete W. (2012). Incorporating a mucosal environment in a dynamic gut model results in a more representative colonization by lactobacilli. *Microb. Biotechnol.* 5 106–115 10.1111/j.1751-7915.2011.00308.x 21989255 PMC3815277

[B279] van Hemert S. Meijerink M. Molenaar D. Bron P. A. De Vos P. Kleerebezem M. (2010). Identification of *Lactobacillus plantarum* genes modulating the cytokine response of human peripheral blood mononuclear cells. *BMC Microbiol.* 10:293 10.1186/1471-2180-10-293 PMC3000848 21080958

[B280] Vankerckhoven V. Huys G. Vancanneyt M. Vael C. Klare I. Romond M.-B. (2008). Biosafety assessment of probiotics used for human consumption: recommendations from the EU-PROSAFE project. *Trends Food Sci. Technol.* 19 102–114 10.1016/j.tifs.2007.07.013

[B281] Vastano V. Salzillo M. Siciliano R. A. Muscariello L. Sacco M. Marasco R. (2014). The E1 beta-subunit of pyruvate dehydrogenase is surface-expressed in *Lactobacillus plantarum* and binds fibronectin. *Microbiol. Res.* 169 121–127 10.1016/j.micres.2013.07.013 24054819

[B282] Ventura M. Jankovic I. Walker D. C. Pridmore R. D. Zink R. (2002). Identification and characterization of novel surface proteins in *Lactobacillus johnsonii* and *Lactobacillus gasseri*. *Appl. Environ. Microbiol.* 68 6172–6181 10.1128/AEM.68.12.6172-6181.2002 12450842 PMC134427

[B283] Verdu E. F. Collins S. M. (2004). Microbial-gut interactions in health and disease. Irritable bowel syndrome. *Best Pract. Res. Clin. Gastroenterol.* 18 315–321 10.1016/j.bpg.2003.11.003 15123072

[B284] Vesterlund S. Paltta J. Karp M. Ouwehand A. C. (2005). Adhesion of bacteria to resected human colonic tissue: quantitative analysis of bacterial adhesion and viability. *Res. Microbiol.* 156 238–244 10.1016/j.resmic.2004.08.012 15748990

[B285] Viaud S. Saccheri F. Mignot G. Yamazaki T. Daillere R. Hannani D. (2013). The intestinal microbiota modulates the anticancer immune effects of cyclophosphamide. *Science* 342 971–976 10.1126/science.1240537 24264990 PMC4048947

[B286] Villena J. Salva S. Aguero G. Alvarez S. (2011). Immunomodulatory and protective effect of probiotic *Lactobacillus casei* against *Candida albicans* infection in malnourished mice. *Microbiol. Immunol.* 55 434–445 10.1111/j.1348-0421.2011.00334.x 21395664

[B287] Vinderola C. G. Medici M. Perdigon G. (2004). Relationship between interaction sites in the gut, hydrophobicity, mucosal immunomodulating capacities and cell wall protein profiles in indigenous and exogenous bacteria. *J. Appl. Microbiol.* 96 230–243 10.1046/j.1365-2672.2004.02158.x 14723684

[B288] Vogel S. N. (2012). How discovery of Toll-mediated innate immunity in *Drosophila* impacted our understanding of TLR signaling (and vice versa). *J. Immunol.* 188 5207–5209 10.4049/jimmunol.1201050 22611247

[B289] Wang C. Wang J. Gong J. Yu H. Pacan J. C. Niu Z. (2011). Use of *Caenorhabditis elegans* for preselecting *Lactobacillus* isolates to control *Salmonella typhimurium*. *J. Food Prot.* 74 86–93 10.4315/0362-028X.JFP-10-155 21219766

[B290] Wang L. Cao H. Liu L. Wang B. Walker W. A. Acra S. A. (2014). Activation of epidermal growth factor receptor mediates mucin production stimulated by p40, a *Lactobacillus rhamnosus* GG-derived protein. *J. Biol. Chem.* 289 20234–20244 10.1074/jbc.M114.553800 24895124 PMC4106339

[B291] Waters C. M. Bassler B. L. (2005). Quorum sensing: cell-to-cell communication in bacteria. *Annu. Rev. Cell Dev. Biol.* 21 319–346 10.1146/annurev.cellbio.21.012704.131001 16212498

[B292] Weiss G. Jespersen L. (2010). Transcriptional analysis of genes associated with stress and adhesion in *Lactobacillus acidophilus* NCFM during the passage through an *in vitro* gastrointestinal tract model. *J. Mol. Microbiol. Biotechnol.* 18 206–214 10.1159/000316421 20559014

[B293] Westermann C. Zhurina D. Baur A. Shang W. Yuan J. Riedel C. (2012). Exploring the genome sequence of *Bifidobacterium bifidum* S17 for potential players in host-microbe interactions. *Symbiosis* 58 191–200 10.1007/s13199-012-0205-z

[B294] Wollowski I. Rechkemmer G. Pool-Zobel B. L. (2001). Protective role of probiotics and prebiotics in colon cancer. *Am. J. Clin. Nutr.* 73 451s–455s. 11157356 10.1093/ajcn/73.2.451s

[B295] Wu R. Sun Z. Wu J. Meng H. Zhang H. (2010). Effect of bile salts stress on protein synthesis of *Lactobacillus casei* Zhang revealed by 2-dimensional gel electrophoresis. *J. Dairy Sci.* 93 3858–3868 10.3168/jds.2009-2967 20655455

[B296] Wu X. Vallance B. A. Boyer L. Bergstrom K. S. Walker J. Madsen K. (2008). *Saccharomyces boulardii* ameliorates *Citrobacter rodentium*-induced colitis through actions on bacterial virulence factors. *Am. J. Physiol. Gastrointest. Liver Physiol.* 294 G295–G306 10.1152/ajpgi.00173.2007 18032474

[B297] Yadav H. Jain S. Sinha P. R. (2008). The effect of probiotic dahi containing *Lactobacillus acidophilus* and *Lactobacillus casei* on gastropathic consequences in diabetic rats. *J. Med. Food* 11 62–68 10.1089/jmf.2006.136 18361739

[B298] Yan F. Cao H. Cover T. L. Whitehead R. Washington M. K. Polk D. B. (2007). Soluble proteins produced by probiotic bacteria regulate intestinal epithelial cell survival and growth. *Gastroenterology* 132 562–575 10.1053/j.gastro.2006.11.022 17258729 PMC3036990

[B299] Yang F. Wang J. Li X. Ying T. Qiao S. Li D. (2007). 2-DE and MS analysis of interactions between *Lactobacillus fermentum* I5007 and intestinal epithelial cells. *Electrophoresis* 28 4330–4339 10.1002/elps.200700166 18004711

[B300] Yoshida E. Sakurama H. Kiyohara M. Nakajima M. Kitaoka M. Ashida H. (2012). *Bifidobacterium longum* subsp. *infantis* uses two different β-galactosidases for selectively degrading type-1 and type-2 human milk oligosaccharides. *Glycobiology* 22 361–368 10.1093/glycob/cwr116 21926104

[B301] Zacarias M. F. Reinheimer J. Forzani L. Grangette C. Vinderola G. (2014). Mortality and translocation assay to study the protective capacity of *Bifidobacterium lactis* INL1 against *Salmonella* Typhimurium infection in mice. *Benef. Microbes* 5 427–436 10.3920/BM2013.0086 24902954

[B302] Zheng Y. Lu Y. Wang J. Yang L. Pan C. Huang Y. (2013). Probiotic properties of *Lactobacillus* strains isolated from Tibetan kefir grains. *PLoS ONE* 8:e69868 10.1371/journal.pone.0069868 PMC3718794 23894554

[B303] Zoumpopoulou G. Foligne B. Christodoulou K. Grangette C. Pot B. Tsakalidou E. (2008). *Lactobacillus fermentum* ACA-DC 179 displays probiotic potential *in vitro* and protects against trinitrobenzene sulfonic acid (TNBS)-induced colitis and *Salmonella* infection in murine models. *Int. J. Food Microbiol.* 121 18–26 10.1016/j.ijfoodmicro.2007.10.013 18077037

[B304] Zyrek A. A. Cichon C. Helms S. Enders C. Sonnenborn U. Schmidt M. A. (2007). Molecular mechanisms underlying the probiotic effects of *Escherichia coli* Nissle 1917 involve ZO-2 and PKCζ redistribution resulting in tight junction and epithelial barrier repair. *Cell Microbiol.* 9 804–816 10.1111/j.1462-5822.2006.00836.x 17087734

